# COVID-19 burden of illness in people who are immunocompromised due to cancer: an expert opinion review

**DOI:** 10.1093/oncolo/oyaf074

**Published:** 2025-06-17

**Authors:** Igor Aurer, Paul Moss, Michel Goldman, Mark Tuthill, Hermann Einsele, Carolina Casañas i Comabella, Samantha James, Katarzyna Borkowska, Fungwe Jah, Sabada Dube, Sarah Klein, Walid Kandeil, Renata Yokota, Antonio Pagliuca, Gkikas Magiorkinis, Sofie Arnetorp, Lennard Lee

**Affiliations:** University Hospital Centre Zagreb, 10000 Zagreb, Croatia; Department of Internal Medicine, Medical School, University of Zagreb, 10000 Zagreb, Croatia; College of Medicine and Health, University of Birmingham, Birmingham, B15 2TT, UK; University Hospitals Birmingham, NHS Foundation Trust, Birmingham, B15 2GW, UK; I3h Institute, Université Libre de Bruxelles, Bruxelles 1050, Belgium; University of Oxford, Manor Hospital/Genesis Care, Oxford, OX4 6LB, UK; Department of Internal Medicine II, Würzburg University Hospital, Würzburg 97080, Germany; EVIDERA, Evidence, Value and Access by PPD, Granta Park Great Abington, Cambridge, CB21 6GQ, UK; EVIDERA, Evidence, Value and Access by PPD, Paris, 94853, France; EVIDERA, Evidence, Value and Access by PPD, Warsaw, 02-672, Poland; Medical Affairs, BioPharmaceuticals Medical, AstraZeneca, Hamburg, 22763, Germany; Vaccines and Immune Therapies Unit, AstraZeneca, Cambridge, CB2 0AA, UK; Medical Affairs, BioPharmaceuticals Medical, AstraZeneca, Groot-Bijgaarden, 1702, Belgium; Vaccines and Immune Therapies, BioPharmaceuticals Medical, AstraZeneca, Baar, 6340, Switzerland; P95 Epidemiology & Pharmacovigilance, Leuven, 3000, Belgium; Stem Cell Transplantation, King’s College London/King’s College Hospital, London, SE5 9RS, UK; Department of Hygiene, Epidemiology and Medical Statistics, Medical School, National and Kapodistrian, University of Athens, Athens 115 72, Greece; Health Economics & Payer Evidence, Vaccines & Immune Therapies, BioPharmaceuticals Medical, AstraZeneca, Gothenburg, 431 50, Sweden; Centre for Immuno-oncology, Nuffield Department of Medicine, University of Oxford, Oxford, OX3 7DQ, UK; Institute of Cancer and Genomic Sciences, University of Birmingham, Birmingham, B15 2TT, UK

**Keywords:** cost of illness, COVID-19, hospitalization, neoplasms, pandemic, vaccination

## Abstract

From the beginning of the pandemic, people with cancer have experienced a high burden from COVID-19 compared with the general population, both in terms of severe COVID-19-related outcomes and reduced health-related quality of life and mental health. This review presents and discusses expert views on the burden of COVID-19 in individuals with cancer throughout the pandemic. The literature suggests that early in the pandemic, people with cancer had a disproportionately high risk of COVID-19-related hospitalization compared with the general population. This trend continued throughout the pandemic, even after the availability of vaccinations (including boosters) and the emergence of less virulent strains. Rates of hospitalization, intensive care unit admission, and mechanical ventilation varied across studies but were all seen to be higher in people with cancer and COVID-19 compared with the general population or those with cancer alone. Moreover, studies indicated worsened quality of life and mental health in these people during the pandemic and lockdown periods compared with prepandemic or postlockdown periods. Although COVID-19 has entered the endemic phase and is no longer a global health emergency, it remains a significant risk for people with cancer. Generally, COVID-19 continues to increase healthcare resource use, impair mental health, and reduce quality of life in this population, highlighting the need for continued real-world studies. Ongoing research is essential to evaluate the impact of COVID-19 on vaccinated people with cancer, particularly those undergoing systemic cancer therapy who may require continued guidance on preventive measures and treatments to mitigate the risk of severe COVID-19.

**Implications for Practice**

Through systematic review of current medical literature and expert input from clinicians, we show the overall heightened burden of COVID-19 among immunocompromised patients, particularly those with cancer, and how COVID-19 continues to impact both individuals and the healthcare community. People with cancer should be kept informed of the reduced protection from vaccines and given guidance on cancer treatment options and what to do if they do contract COVID-19. Clinicians treating this population should continue to be vigilant regarding prevention and protection measures against COVID-19 and stay aware of the continuing impact of COVID-19, as well as other infectious diseases.

## Introduction

People living with cancer represent a diverse population with varied risks of COVID-19 and its complications.^1,2^ People with hematological malignancies or active and/or metastatic cancers and those receiving cancer treatments have been at particularly high risk for severe COVID-19 outcomes,^3^ including COVID-19-related death, since the beginning of the COVID-19 pandemic.^4-7^ While vaccination and the emergence of less pathogenic SARS-CoV-2 strains have reduced mortality in the general population,^8^ vaccinated people with cancer are still at a disproportionately increased risk of severe COVID-19 outcomes compared with vaccinated people without cancer.^2,9,10^ People with cancer have been shown to have higher rates of hospitalization (55.2% vs 28.9%), intensive care unit (ICU) admissions (25.4% vs 11.7%), and death (30-day mortality, 13.4% vs 1.6%) from COVID-19 vs the general population.^11^ These individuals are also prone to prolonged viral shedding, extended SARS-CoV-2 infection, and COVID-19 relapse,^12-16^ all of which are associated with an increased risk of complications from COVID-19.^17^

Severe COVID-19 outcomes in people with cancer may be attributed to systemic immunosuppression caused by the cancer itself or cancer treatments, which increases susceptibility and physiological response to infection.^18-20^ Treatment-related systemic immunosuppression has also been associated with impaired serologic responses to COVID-19 vaccines in people with cancer,^21-23^ evidenced by lower seroconversion rates,^24,25^ decreased neutralization activity,^26,27^ and early antibody waning.^28^

Practicing social avoidance and protective behaviors was initially recommended to minimize the risk of COVID-19 in immunosuppressed people and is still practiced by many with an inadequate response to vaccination.^29^ However, these practices have had detrimental effects on health-related quality of life (HRQoL), with people at high risk and their caregivers reporting increased feelings of isolation and feeling “left behind” as the general population returns to pre-COVID living.^30^

COVID-19 has affected people with cancer and the global medical community across all aspects of the disease. During the first year of the pandemic, 20% of people with cancer experienced delays in routine appointments, screening tests, and blood tests,^31^ and COVID-19 also led to delays and interruptions in treatment.^32,33^ Delayed or suboptimal cancer treatment has been attributed to high COVID-19-related mortality rates in people with hematological cancers; secondary bacterial or fungal infections may also have contributed.^34^ These delays also had an impact on the mental well-being of people with cancer, which was sometimes exacerbated by poor communication from healthcare professionals.^32,35^

As the pandemic transitions to an endemic phase and the impact of COVID-19 has decreased among the general population, it is important to examine and highlight the continuing burden of SARS-CoV-2 on people with cancer. In this expert review, a group of oncology, immunology, virology, and epidemiology experts used published literature and their clinical experience to demonstrate the ongoing burden of COVID-19 experienced by people with cancer.

## Methods

Targeted searches were conducted in Embase, Medline, PsycInfo, and EconLit, directly and using OvidSP (Tables S1–S4), for global scientific publications (including conference abstracts) about the burden of COVID-19 in people with immunocompromising conditions (Table S5). Additional filtering for this review was then conducted to focus on those with solid tumors and hematological malignancies or people receiving chimeric antigen receptor T-cell therapy, hematopoietic stem cell transplants, chemotherapies, or tumor necrosis factor inhibitors. Only real-world studies conducted in humans and published in English were included; clinical trials were excluded. Searches were conducted throughout the development of this review, the most recent in November 2023.

To provide a comprehensive look at the burden throughout the pandemic and specific context among each distinct COVID-19 period, this review only includes studies that reported data from specific time periods, broadly classified as early pandemic (2020), mid-pandemic (2021), and Omicron (late 2021-2022). Studies included data spanning the beginning of the pandemic to the Omicron era (up to the end of December 2022).^2^ The specific SARS-CoV-2 variant was rarely reported; therefore, the variant was inferred based on the study enrollment period.

### Real-world clinical burden of COVID-19 in people with cancer

#### Overview of clinical outcomes

COVID-19 may be associated with treatment modification and increased risk of relapse and hospitalization, substantially increasing healthcare resource utilization (HCRU) and overall burden on QoL. The current evidence confirms the increased risk of developing severe COVID-19 and greater mortality for people with cancer compared with the general population,^10^ which continued even after vaccination and during Omicron.^10,36^

In 2020-2021, the estimated risk of death for people with hematological malignancies diagnosed with COVID-19 was 34%^37^; the risk of death from COVID-19 was 1.83 times and 2.85 times higher in those with solid tumors or hematological cancer, respectively, compared with those without cancer.^38^ This increased susceptibility continued during Omicron.^2^

Clinical observations suggest that patients with hematological malignancy and those currently undergoing chemotherapy, stem cell transplants, or intensely immune suppressive therapies remain at the greatest risk among people with cancer.

#### Summary of clinical burden of COVID-19 in people with cancer

We have seen in the clinic that people with cancer who have mild symptoms at onset when infected with SARS-CoV-2 can later develop serious and prolonged disease. Since the Omicron variant became predominant, high-risk people who develop COVID-19 not only remained susceptible to prolonged infection, which interfered with appropriate treatment of their underlying disease, but continued to die from COVID-19.^2^

### Economic and outcomes-based burden of COVID-19 in people with cancer

Only one study within our designated timeframe reported COVID-19-related costs among people with cancer with COVID-19. In a large, multicenter retrospective database study from the United States (April 2020-March 2022), the mean cost of COVID-19-related hospitalizations per patient was $30 671 (standard deviation: $68 253) among 3820 people with cancer.^39^

#### Healthcare resource utilization

Clinical/service evaluation studies were identified that included information about COVID-19-related HCRU (eg, hospitalization or ICU rates) (Table 1 and Table S6); however, data were not identified regarding HCRU related to “long COVID-19.” Rates of hospitalization, ICU admission, and mechanical ventilation varied across studies (Table 1 and Table S6) and were generally higher in people with hematological cancer vs those with solid tumors.

**Table 1.** Healthcare resource utilization during mid-pandemic and the Omicron wave.

Study date/country	Population	Vaccination status	Hospitalizations/ICU admission/MV, *n* (%)	Risk factor group comparisons
**Mid-pandemic/Alpha/Delta wave (late 2020–2021)**				
Jun-Oct 2021 Colombia^48^	Adults with cancer & COVID-19 (*n* = 896)	Unvaccinated (*n* = 724)	307 (42)/-/61 (8)	–
		Vaccine series complete (*n* = 172)	50 (29)/-/8 (5)	
Dec 2020-Nov 2021 England^9^	Adults with solid cancers & COVID-19 (*n* = 25 625)	Vaccinated (2 doses)	4065 (16)/-/-	–
	Adults with hematological malignancies & COVID-19 (*n* = 3725)		1225 (33)/-/-	–
Apr-May 2021 Europe^49^	Adults with CLL & COVID-19 (*n* = 793)	Not reported	593 (75)/162 (20)/-	–
2021-2022 USA^50^	Adults with cancer & COVID-19 (*n* = 2486)	Unvaccinated (*n* = 1537)	N/A (57)/N/A (16)	• Hospitalization: Increased age (per 10 years increase) (aOR, 1.34); progressing malignancy (vs not) (aOR, 2.15); and ECOG PS ≥ 2 (ref 0) (aOR, 3.31) • ICU/MV: Increased age (per 10 years increase) (aOR, 1.20); progressing malignancy (vs not) (aOR, 1.55); and ECOG PS ≥ 2 (ref: 0) (aOR, 2.29)
		Vaccinated, 2 doses (*n* = 564)	N/A (50)/N/A (12)	
		Vaccinated, 3 doses (*n* = 385)	N/A (38)/N/A (8)	
Feb-Nov 2020 Europe^7^	Adults with cancer & COVID-19 (*n* = 1075)	Vaccinated (*n* = 152; 14.1%)	437 (41)/-/-	–
Jul-Aug 2021 France^53^	Adults with cancer (*n* = 2 368 556)	Vaccinated (2 doses or single dose with prior COVID-19)	1604 (0.07)/-/-	Hospitalization • Active lung cancer (HR, 3.45) • Other active cancers^b^ (HR, 3.03)
**Omicron wave (Dec 2021-2022)**				
Dec 2021-Feb 2022 UK^51^	Adults with hematological malignancies and breakthrough infection (*n* = 57)	Received 4th dose during study	6 (11)/-/-	–
Dec 2021-Mar 2022 (follow-up Jun 2022) England^10^	Adults with hematological malignancies & SARS-CoV-2+ (*n* = 8389)	Not reported by population	741 (9)/-/-	Hospitalization: Women • Chemotherapy in the last 12 months^c^ (vs none) ◦ Grade A (aHR, 3.33); Grade B (aHR, 4.76); and Grade C (aHR, 3.69) • Blood cancer (aHR, 1.96) • Respiratory cancer (aHR, 1.83) Hospitalization: Men • Chemotherapy in the last 12 months (vs none) ◦ Grade A (aHR, 2.41); Grade B (aHR, 3.90); and Grade C (aHR, 4.47) • Blood cancer (aHR, 1.97) • Respiratory cancer (aHR, 1.39)
	Adults with respiratory cancer & SARS-CoV-2 + (*n* = 1743)		167 (10)/-/-	–
Jan-Dec 2022 England^2^	People aged ≥ 12 years (*N* = 6 060 635)	Vaccinated with ≥ 3 doses by study start	14 365 (< 1)/255 (< 1)/-	Hospitalization and ICU admission respectively (all aIRRs vs without condition) • Solid tumor (≤ 5 years prior) (aIRRs: 1.40; 1.22) ◦ With active treatment (≤ 6 months prior) (aIRRs: 3.65; 2.85) ◦ With recent treatment (6-12 months prior) (aIRRs: 2.23; 2.27) ◦ With distant treatment (> 12 months prior) (aIRRs: 1.33; 0.43) • Hematological malignancy (≤ 5 years prior) (aIRRs: 3.62; 13.58) ◦ With active treatment (≤ 6 months prior) (aIRRs: 10.63; 38.33) ◦ With recent treatment (6-12 months prior) (aIRRs: 5.80; 28.43) ◦ With distant treatment (> 12 months prior) (aIRRs: 3.03; 8.00) ◦ CLL, nHL, MM, acute leukemia (≤ 2 years prior) (aIRRs: 4.96; 20.55)
	People aged ≥ 12 years with solid tumors (*n* = 265 960)		1890 (1)/<10 (< 1)/-	
	People aged ≥ 12 years with hematological malignancies (*n* = 50 665)		865 (2)/ 20 (< 1)/-	
Dec 2021-Feb 2022 France^52^	Adults with hematological malignancies & COVID-19 (*n* = 57)	94.7% received full vaccination schedule (3-4 doses)	13 (23)/-/11 (19)	Hospitalization • Negative or weak serology in hospitalized vs nonhospitalized people (*P < *.05)
Feb-Nov 2022 Europe^7^	Adults with cancer & COVID-19 (*n* = 365)	Vaccinated^a^ (*n* = 133; 36.4%)	86 (24)/-/-	–

A dash (–) in the cell indicates data are not reported in the study.

^a^Defined as 2 doses of Pfizer–BioNTech, mRNA-1273 (Moderna), and ChAdOx1-S (Oxford–AstraZeneca) or single dose of Ad26.COV2.S (Johnson & Johnson) vaccine.

^b^Other cancer refers to all cancers except female breast cancer, colorectal cancer, lung cancer, and prostate cancer.

^c^Chemotherapy group A, B, or C is based on the risk of grade 3 or 4 febrile neutropenia (per Common Terminology Criteria for Adverse Events version 4) or lymphopenia.

Abbreviations: aHR, adjusted hazard ratio; aIRR, adjusted incremental rate ratio; aOR, adjusted odds ratio; CLL, chronic lymphocytic leukemia; ECOG PS, Eastern Cooperative Oncology Group performance status; HR, hazard ratio; ICU, intensive care unit; MM, multiple myeloma; MV, mechanical ventilation; N/A, not available; nHL, non-Hodgkin’s lymphoma; OR, odds ratio.

##### Early pandemic (2020)

 A substantial amount of data has been published regarding hospitalization rates during the beginning of the pandemic (before vaccination rollout); in people with cancer during this time, hospitalization rates of up to 88% were reported (Table S6). Hospitalization rates were approximately 4 times higher in people with cancer and COVID-19 vs people with cancer and no COVID-19, and approximately 1.5-2 times higher vs people with COVID-19 who did not have cancer.^40-42^ In one study, COVID-19 was attributed to an excess rate of hospitalization of 12% compared with people with cancer but without COVID-19.^43^ Where reported, the median hospitalization length of stay (LOS) was 6 days (range, 0-135) in people with COVID-19 and cancer,^44^ and mean LOS was significantly higher for people with COVID-19 and cancer vs people with COVID-19 but without cancer (13.1 vs 11.5 days; *P* < .001).^38^

Intensive care unit admission rates of up to 45% were reported during the prevaccination period (Table S6).^38,42,45,46^ Intensive care unit admissions were 1.1-2.5 times higher in people with cancer and COVID-19 compared with people without cancer but with COVID-19, and an excess rate of 12% of ICU admissions was attributed to COVID-19 in people with cancer compared with people with cancer but without COVID-19.^38,42,43^ Length of stay in the ICU was similar in people with cancer and COVID-19 vs people with COVID-19 but without cancer (10.9 vs 11.1 days; *P* = .526).^38^

Rates of mechanical ventilation were not reported by many of the studies identified. Rates ranged from 5% to 27%,^38,45,47^ and COVID-19 was associated with an excess rate of mechanical ventilation of approximately 7% compared with people with cancer but without COVID-19.^43^

##### Mid-pandemic (late 2020-late 2021)

 Mid-pandemic (after COVID-19 vaccinations were available) rates of hospitalization due to COVID-19 of up to 75% were reported in people with cancer (Table 1).^7,9,48-50^ Hospitalization rates were higher for people with hematological malignancies vs solid tumors,^9^ and where vaccination status was compared, people with more doses of COVID-19 vaccine had lower rates of hospitalization vs those with fewer or no doses.^48,50^ However, even after receiving the full series of vaccinations (defined as doses available at time of study), rates of hospitalization were reduced by only one-third compared with those who were not vaccinated.^48,50^

Intensive care unit admissions and rates of mechanical ventilation were also lower in people with more doses of the vaccine compared with those who were not vaccinated.^48,50^

##### Omicron era (late 2021-study end)

During Omicron, people with cancer had higher rates of vaccination and lower hospitalization rates than during 2020-2021 (9%-24%; Table 1).^7,10,51,52^ In people who had received 3 doses of COVID-19 vaccination, hospitalization rates were more than 3 times higher among people with cancer vs the general population and twice as high among people with hematological malignancies vs those with solid tumor cancers.^2^

Similar trends were seen in ICU admissions and rates of mechanical ventilation (Table 1).^2,52^

##### Postpandemic (endemic; 2023 onwards)

At the time of writing, literature reporting HCRU in the endemic period was not available. Based on the clinicians’ experience, the rate of infection with SARS-CoV-2 remains high, but outcomes have considerably improved for people with cancer—with far fewer COVID-19 hospitalizations and ICU admissions vs during the pandemic. Some of this may be due to immunity acquired from vaccination and/or SARS-CoV-2 infection in those who were diagnosed after the pandemic. Despite this, in our clinical experience, people with cancer continue to face a higher risk of HCRU compared with the general population.

##### COVID-19 risk factors for HCRU

Throughout the pandemic, numerous studies identified risk factors associated with increased rates of hospitalization, ICU admission, and mechanical ventilation (Table 1 and Table S6). Risk factors included receiving cancer therapy within 12 months before COVID-19 and/or having hematological malignancy, progressive cancer, and an Eastern Cooperative Oncology Group (ECOG) performance status ≥2. Overall risk factors generally remained consistent throughout the pandemic.^2,10,40-42,46,53^

### Impact on quality of life

Most studies reporting the personal burden of people living with cancer covered Europe and the UK. Studies varied in sample size and utilized a wide range of patient-reported outcomes and HRQoL scales (Tables 2 and 3 and Table S6).

**Table 2.** Overview of the HRQoL results in people with cancer.

Study date/country	Population	Vaccination/infection status	Instrument/Comparison	QoL results
**Early pandemic (early to mid-2020): prevaccination**				
Jan 2019-Aug 2020 USA^59^	People with multiple myeloma (*n* = 199)	•Unvaccinated •Infection status unknown	EQ-5D-5L/pandemic vs prepandemic	• Overall health state decreased by 16% pre- to postpandemic • Worsening in all 5 domains prepandemic to postpandemic
May 2020-Jun 2020 Denmark^55^	Adults with hematological cancers (*n* = 2239)	•Unvaccinated •Infection status unknown	EORTC QLQ-C30/pandemic vs prepandemic	• Mean global health status during pandemic suggests reduced but acceptable QoL • Being concerned about contracting COVID-19 lowered QoL, emotional, and social functioning scores • Having multiple myeloma or multiple hematological diseases associated with decreased QoL (ref: lymphoma)
May 2020 Denmark^57^	Adults with solid cancer (*n* = 4571)	•Unvaccinated •COVID-19: 0.4%	EORTC QLQ-C30/pandemic vs prepandemic	• Mean global health score during pandemic: 71.3 • No significant differences in score between this study and a 2017 study in adults with any cancer (Barometer study cohort; mean score, 74.4) • Concerns over contracting COVD-19 associated with decreased QoL
Mar 2020-Sep 2020 France^60^	Nonmetastatic breast cancer or gynecological cancer (*n* = 125)	•Unvaccinated •COVID-19: 3.4%	EORTC QLQ-C30/lockdown vs postlockdown	• QoL was significantly higher postlockdown vs during lockdown
			SF-12/lockdown vs postlockdown	
Jun 2020 Italy^54^	Adults (30-70 years) with or without any cancer on active treatment (*n* = 312)	•Unvaccinated •COVID-19: none infected	SF-12/cancer (*n* = 111) vs general population (*n* = 201)	People with cancer experience the following vs the general population: • Significantly lower levels of fear of COVID-19, cancer, and other diseases • Significantly lower physical component scores • Significantly higher mental component scores
**Mid-pandemic/Alpha/Delta wave (late 2020-2021)**				
Jul 2021 Germany^58^	Adults with pancreatic cancer who had undergone surgery (*n* = 26)	•Vaccinated: 96% •Infection status unknown	EORTC QLQ-C30/ pandemic vs prepandemic	• QoL not significantly reduced during the pandemic vs prepandemic • Role and emotional functioning scores decreased during pandemic; emotional attributed to pandemic • Vaccination improved QoL: 48%
Apr 2021-May 2021 Vietnam^56^	Adults with solid cancer actively receiving chemotherapy (*n* = 270)	•Majority unvaccinated •COVID-19: 0	EORTC QLQ-C30/cancer stage, type, treatment	• Mean global health status during pandemic: 56.7 • Associated with worse HRQoL: ◦ High concerns about contracting COVID-19 ◦ Delaying chemotherapy ◦ Not controlling cancer well ◦ More concern about either cancer or COVID-19 over the other

Shading indicates decreased QoL during pandemic/in people with cancer vs relevant comparator.

Abbreviations: EORTC QLQ-C30, European Organization for Research and Treatment of Cancer Quality of Life Questionnaire Core 30; HRQoL, health-related quality of life; QoL, quality of life; SF-12, 12-item Short Form Health Survey.

**Table 3.** Overview of the mental health results in people with cancer during mid-pandemic and the Omicron wave.

Study date/country	Population	Vaccination/infection status	Domain/instrument/comparison	Mental health results
**Mid-pandemic/Alpha/Delta wave (late 2020-2021)**				
Sep 2021-Nov 2021 Germany^70^	Adults with any cancer (*n* = 438)	• Vaccinated: 95.5% • Infected status unknown	Anxiety/GAD-7/none	• Mean anxiety score: 5.1 (moderate anxiety) • Negative correlation between vaccination status and obsessive thinking about COVID-19
			Distress/FCV-19S/none	
Jan 2021-Feb 2021 Canada^68^	Adolescents and young adults (< 40 years) with any cancer (*n* = 805)	• Vaccination and infection status unknown	Distress/K-10/pre- vs during pandemic	• A significantly higher proportion reported moderate-to-severe distress during pandemic vs prepandemic • Employment impact and hematological cancer associated with higher distress
Jan 2021-Feb 2021 Canada^65^	Adults < 40 years with any cancer (*n* = 805)	• Vaccination and infection status unknown	Distress/K-10/none	• Distress, 88.2% (moderate-severe: 68.0%); insomnia, 38.8%; and lonely, 52.0% • Experiencing distress during pandemic largest contributor to increased insomnia
			Insomnia/ISI/none	
			Loneliness/UCLA-3/none	
Jan 2021-Feb 2021 Canada^66^	Adults ≤ 40 years old with any cancer (*n* = 805)	• Vaccination and infection status unknown	Loneliness/UCLA-3/none	• Lonely, 52%; loneliness was most likely to be reported in people: undergoing cancer therapy, had a self-disclosed prepandemic mental health condition, or was not in a relationship • People living in rural/remote locations less likely to report loneliness
Mar 2020-Feb 2021 Mexico^62^	Adults with thoracic neoplasm with and without COVID (*n* = 144)	• Vaccination status unknown • COVID-19: 52.5%	Depression/DASS-21/none	• Depression and distress, 18% (each); anxiety, 31% • People with distress or depression had higher odds of experiencing treatment delays than people without
			Distress/DASS-21/none	
			Anxiety/DASS-21/none	
Feb 2019-Dec 2021 Spain^71^	Adults with advanced, unresectable cancer (*n* = 636)	• Vaccination and infection status unknown	Stress/BRCS/none	• High levels of resilience and distress • Resilience correlated positively with spirituality • Psychological stress correlated negatively with resilience and spiritual well-being
			Distress/BSI/none	
Feb 2020-May 2021 Spain^61^	Adults with newly diagnosed advanced cancer (*n* = 401)	• Vaccination and infection status unknown	Depression/BSI/none	• Clinically significant symptoms of depression: 35% • Clinically significant symptoms of anxiety: 36% • Being female, having a preoccupation about cancer, feeling hopelessness, and having a younger age for anxiety were associated with increased levels of anxiety or depression
			Anxiety/BSI/none	
Apr 2020-May 2021 France^82^	Adults with any cancer (*n* = 386)	• Vaccination and infection status unknown	Distress/IES-R/first lockdown vs release period	• Low level of PTSD throughout the pandemic • Levels of PTSD increased during lockdown periods • PTSD symptoms associated with increased insomnia and cognitive complaints
			Distress/IES-R/second lockdown vs release period	
**Omicron wave (Dec 2021-2022)**				
Apr 2021-Mar 2022 USA^69^	Adults with cancer undergoing outpatient palliative care (*n* = 200)	• Vaccinated: 85.2% • COVID-19: 16.7%	Distress/COVID-19 distress survey/pre- vs during pandemic	• Worse levels of distress vs before pandemic: 40% ◦ Majority reporting worse distress fully vaccinated at time of study • Gastrointestinal, genitourinary, and head and neck cancers reporting worsened distress: > 70% ◦ Significant predictors of increased distress: Age, worsened social isolation, negative attitude to staying at home, and depression at baseline • Coping strategies significantly associated with distress included snacking and distracting themselves
Nov 2021-Feb 2022 Iran^72^	Adults with cancer undergoing chemotherapy (*n* = 155)	• Vaccination status unknown • COVID-19 history: 46.5%	Spiritual well-being/Ellison’s spiritual well-being scale/none	• Moderate levels of COVID-19 anxiety, spiritual well-being, and resilience • Increased spiritual well-being significantly associated with increased resilience • Lower spiritual well-being significantly associated with increased COVID-19 anxiety • Low resilience correlated with COVID-19 anxiety

Shading indicates poorer mental health during pandemic/in people with cancer vs relevant comparator.

Abbreviations: BRCS, Brief Resilient Coping Scale; BSI, Brief Symptom Inventory; DASS-21, Depression Anxiety Stress Scale 21; FCV-19S, Fear of COVID-19 Scale; GAD-7, General Anxiety Disorder-7; HADS, Hospital Anxiety and Depression Scale; IES-R, Impact of Event Scale—Revised; ISI, Insomnia Severity Index; K-10, Kessler Psychological Diagnosis System 38; PROMIS-29, Patient-Reported Outcomes Measurement Information System-29; PTSD, post-traumatic stress disorder.

#### HRQoL

Among studies that reported on HRQoL during the pandemic, there were variations in findings (Table 2). In one study, people with solid and hematological malignancies indicated worse physical HRQoL but better mental well-being than the general population,^54^ while in another study, people with hematological cancers reported acceptable levels of overall quality of life (QoL) during the pandemic.^55^ For people with solid tumors who reported low scores for HRQoL during the pandemic, HRQoL did not differ significantly by disease stage, cancer location, or previous treatment.^56^ In general, concerns regarding contracting COVID-19 were associated with decreased HRQoL.^55-57^

Studies evaluating HRQoL between the prepandemic and pandemic phases also showed mixed results. One study reported lower HRQoL of partially vaccinated people with pancreatic cancer during the pandemic than prepandemic,^58^ while a study of people with solid or hematological malignancies found no clinically significant differences in HRQoL scores between prepandemic and pandemic phases.^57^ In another study, people with multiple myeloma reported improvement in overall health state from prepandemic to the pandemic phase.^59^ Further, people with solid tumors reported better HRQoL postlockdown vs during lockdown in both physical and mental domains.^60^

#### Mental health

Overall, levels of depression, anxiety, stress, and distress were higher in people with cancer during the pandemic compared with prepandemic or with the general population (Table 3 and Table S6). Being female, being worried about cancer, feeling hopelessness, and experiencing treatment delays were associated with increased levels of depression and anxiety/distress.^61-63^

In a survey of people with hematological cancers during the early phase of the pandemic, symptoms of generalized anxiety were frequent, with 16% of people presenting mild symptoms of anxiety and 5% with moderate-to-severe symptoms.^55^ In people with thoracic neoplasms, 31% reported anxiety and 18% both depression and distress during the pandemic.^62^ Anxiety was also reported by 31% of people with solid tumors and hematological malignancies during the pandemic.^63^ Clinical observations align with these findings, with people with hematological malignancies showing increased rates of mental health issues, in part due to shielding, compared with before the pandemic.

A survey of people with solid tumors found that mean anxiety scores significantly increased during the pandemic period vs prepandemic.^64^ In people with nonmetastatic breast or gynecological cancer, a significant reduction in depression, but not anxiety, was observed after lockdown vs during lockdown.^60^ Interestingly, in a survey of people with solid tumors and hematological malignancies, mental health in both prepandemic and pandemic periods was comparable (median [interquartile range] K10 scores: 19 [14-25] vs 19 [14-25]; *P* = .919), but only when treatment delays were ≤7 days.^35^

Adolescents and young adults with solid tumors and hematological malignancies during the pandemic showed indications of subclinical insomnia,^65^ with experiencing distress during the pandemic the largest contributor to increased insomnia.^65^ Loneliness was also reported by 52% of adolescents and young adults and was most likely to be reported in people who were undergoing cancer therapy, had a self-disclosed prepandemic mental health condition, or were not in a relationship.^65,66^

Low levels of post-traumatic stress disorder were experienced by most (85%) people with solid and hematological malignancies during the pandemic.^63^ Treatment delays and fear of going to the hospital because of the risk of contracting COVID-19 were associated with post-traumatic stress disorder.^35,63^

High levels of distress were also reported throughout the pandemic, including during Omicron. Adults with solid tumors and hematological malignancies reported that distress significantly increased between early and later in the pandemic.^67^ Similar trends were found in a survey of adolescents and young adults with cancer.^68^ In one study, 40% of people with advanced cancer had increased levels of distress during the pandemic vs prepandemic. Distress was reported by >70% of those with gastrointestinal, genitourinary, and head and neck cancers. The majority of those reporting increased distress were fully vaccinated at time of study. Older age, greater social isolation, a negative attitude about staying at home, and depression at baseline were all significant predictors of increased distress.^69^

In contrast to these results, a survey of partially vaccinated adults with cancer showed participants were not highly concerned about COVID-19.^70^ People with cancer also reported good resilience during the pandemic,^71^ which was increased by spiritual well-being but decreased by psychological stress. Relationships between resilience and spiritual well-being were also examined during Omicron. People undergoing chemotherapy reported moderate levels of COVID-19-related anxiety, spiritual well-being, and resilience. Increased spiritual well-being was significantly associated with increased resilience, while lower spiritual well-being and low resilience were associated with increased COVID-19-related anxiety.^72^

To date, most of the published information regarding mental health in this population represents the time before Omicron. Data from Omicron and beyond are needed to assess and address the ongoing negative impact of COVID-19 on the mental health of people with cancer.

In the clinic, we are now seeing that people with cancer are no longer reporting an impact of COVID-19 avoidance measures on QoL or mental health. They are becoming much more confident about engaging in social activities, and many have reassurance that their vaccination status offers considerable protection. There is anecdotal evidence that uptake of vaccine booster doses is declining, although this may be in part due to ambivalence or vaccine fatigue.

## Discussion

COVID-19 has had a disproportionate impact on people with cancer due to increased risk of infections, hospitalization, and death compared with those without cancer. This increased risk persisted even after vaccination and during Omicron. People with cancer also generally experienced decreased mental health and HRQoL during the pandemic compared with prepandemic or vs those without cancer. Our combined clinical experience suggests that outcomes in people with cancer continue to be at increased risk of severe COVID-19. Although disruptions to cancer clinics have declined, clinicians are still reporting treatment disruptions due to COVID-19 illness.

COVID-19 vaccines have reduced the risk of COVID-19-related hospitalization and mortality; therefore, vaccinations need to be prioritized for those with cancer. However, clinicians and people with cancer should be mindful that the effectiveness of vaccines varies by type of cancer and treatment.

Even after vaccination, for people with cancer, COVID-19 continues to have a considerable impact on HCRU, especially hospitalizations, which have generally been highest among people with hematological malignancies, particularly those undergoing active treatment.^2^ However, this evidence fails to provide the full picture of the indirect potential burden of hospitalizations due to underlying disease progression from treatment interruptions, the increased treatment costs, and other associated issues such as the burden for caregivers. COVID-19 is a new addition to the list of comorbid conditions that worsen cancer patient burden. It is well understood that comorbid, noncommunicable diseases such as diabetes increase mortality risk, prolong hospital stays, and significantly lower QoL vs cancer alone.^73-75^ As a result, careful coordination among oncologists and other physicians involved in patient care is needed to manage, mitigate, or prevent comorbidities. Such coordination is also relevant when considering infectious disease comorbidities like COVID-19 or influenza.^76-78^ Patients with cancer and COVID-19 are more likely to be admitted to the ICU (29.4% vs 24.7%; odds ratio [OR] 1.31 [95% CI 1.00-1.73]; *P* < .05) and have higher mortality risk (34.9% vs 17.9%; OR 2.45 [95% CI 1.81-3.32]; *P* < .01) compared with those with influenza.^79^ These data further underscore the importance of vaccinations and practicing avoidance and protective measures to mitigate and prevent infectious comorbid conditions.

Most studies that evaluated HRQoL showed that COVID-19 had a negative impact. People with cancer generally reported increased rates of anxiety/depression and lower HRQoL during the pandemic/lockdowns vs prepandemic/postlockdown.^55-58^ However, results were heterogeneous, and a variety of instruments were used to assess both HRQoL and mental health, making qualitative comparisons among studies difficult. In addition, only 2 studies used COVID-specific patient-reported outcomes.^69,70^ Consistent HRQoL and mental health instruments focusing on COVID-specific outcomes should be considered as part of future studies.

### Limitations

Among the studies reviewed here, pandemic-related lockdown, avoidance and protective behaviors, and social distancing generally had a meaningful impact not only on people with cancer but also on their caregivers.^30^ This review did not identify any information on the impact of COVID-19 on caregivers of people with cancer, although personal stories and observations from the clinic indicate caregivers are still practicing COVID-19 avoidance and protective behaviors and report a reduced QoL as a result.^80^ Additionally, data surrounding the personal and economic impact of continuing to practice avoidance and protective behaviors in the postlockdown era are limited. This represents a substantial gap in the COVID-19 evidence base.

Only one study reporting HCRU costs in people with cancer and COVID-19 was identified. More real-world studies exploring healthcare costs are needed to provide an expanded view of the impact of COVID-19 on people with cancer.

This review focused on the clinical, economic, and personal burden of COVID-19 in people with cancer and does not focus on COVID-19-related mortality. However, as mortality remained increased in people with cancer vs the overall population during Omicron,^2^ this is also an important outcome and warrants further research. As with hospitalizations, recent treatment, recent chemotherapy, hematological malignancy, and ECOG performance status ≥2 are all risk factors for COVID-19-related mortality.^2,10,81^

Many studies in this review had small sample sizes, highlighting the need for real-world studies with larger populations to better assess HCRU outcomes and economic impact. Another limitation is that while conference abstracts included in this review provided additional valuable preliminary insights, their unvalidated and incomplete nature requires cautious interpretation.

## Conclusion

The literature and clinician experiences reported in this expert review highlight the historic and ongoing burden of COVID-19 in people with cancer, despite the general population shifting to a more endemic/postpandemic approach with the lifting of preventive health measures. Individuals with hematological cancers and/or receiving active cancer treatment continue to be particularly at increased risk for COVID-19, despite available vaccines and high rates of vaccination. Continued vigilance in people with cancer is important to protect against COVID-19, including keeping up with vaccinations, practicing avoidance and protective measures, staying aware of symptoms, and seeking appropriate COVID-19 treatment. Unlike influenza, the seasonal fluctuation of COVID-19 is not fully clear; therefore, people with cancer must always remain vigilant.

Additional studies conducted during Omicron and beyond are vital to understand and mitigate the continuing impact of COVID-19 on people with cancer.

## Supplementary Material

oyaf074_suppl_Supplementary_Tables_1-6

## Data Availability

No new data were generated or analyzed in support of this research.
