# Supplementary material for: COVID-19 burden of illness in people who are immunocompromised due to cancer: an expert opinion review
Source: Oncologist. 2025 Jun 17;30(6):oyaf074. doi: 10.1093/oncolo/oyaf074 (PMC12200237; doi:10.1093/oncolo/oyaf074)
Supplement: oyaf074_suppl_Supplementary_Tables_1-6 [file oyaf074_suppl_supplementary_tables_1-6.pdf]

Supplementary Material

COVID-19 Burden of Illness in People Who Are Immunocompromised Due to Cancer: An Expert Opinion Review

Aurer I, Moss P, Goldman M, et al.

Table of Contents

Supplemental Table 1. Embase search strategy. 2

Supplemental Table 2. Medline search strategy. 7

Supplemental Table 3. PsycInfo search strategy. 11

Supplemental Table 4. EconLit search strategy. 15

Supplemental Table 5. Base immunocompromised population considered in this review article. 18

Supplemental Table 6. Healthcare resource utilization and mental health results during early pandemic. 19

References 22

Supplemental Table 1. Embase search strategy.

No.	Search Terms
1	(covid-19 or covid19 or corona-virus or sars-cov-2 or sars-cov2 or coronavirus disease or ncov or n-cov or delta or omicron).ti.
2	asthma$.ti.
3	(bronchiectasis or bronchiectasia or bronchoectasia).ti.
4	(cancer or neoplasm$ or leuk?emia or lymphoma or mesothelioma or myeloma or melanoma or carcinoma or adenocarcinoma or sarcoma or hemangioma or schwannoma$ or teratoma$ or tumo?r or tumo?rs).ti.
5	(brain angiopathy or brain circulation failure or brain vascular disease or brain vasculopathy or cerebral small vessel disease$ or cerebral vascular disease or cerebral vascular disorder or cerebral vascular disturbance or cerebral vascular lesion or cerebral vasculopathy or cerebrovascular damage or cerebrovascular disorder$ or cerebrovascular lesion or cerebrovascular pathology or cerebrovascular syndrome or basal ganglion h?emorrhage or bow hunter syndrome$ or brain hemangioma or brain hematoma or ((((brain or cerebral or corpus callosum or intracranial or intracerebral or periventricular or posterior fossa) adj2 (h?emorrhage or h?emorrhagic)) or ((brain or cerebral or cerebrovascular or hemisphere) adj2 infarct$) or ((brain or cerebral or cerebrovascular or cerebri or neural) adj2 (isch?emia or arterial insufficiency or circulation disorder$ or blood flow disorder or circulation disorder or circulatory disorder or encephalopathy)) or brain vasospasm or ischemic encephalopathy or transient ischemic attack or carotid artery disease$ or carotid arterial disease or carotid arteriopathy or carotid disease or (carotid artery adj2 (aneurysm or anomaly or bruit or calcification or injury or obstruction or atherosclerosis)) or moyamoya disease or cerebral artery disease$ or cerebrovascular accident or stroke or apoplexia or apoplexy or ((brain or cerebral or cerebrovascular) adj2 (accident or attack or insult or apoplexia or failure)) or cerebrovascular malformation or brain arteriovenous malformation or vein of galen malformation or hypophysis apoplexy or intracranial aneurysm or (brain or cerebral or cerebrovascular or intracranial)) adj2 (obstruction or occlusion or thrombosis or phlebothrombosis or thromboembolism or thrombosis or occlusive)) or melas syndrome or occlusive cerebrovascular disease or ocular isch?emic syndrome or posterior reversible encephalopathy syndrome or vertebrobasilar insufficiency or Wallenberg syndrome).ti.
6	(((chronic kidney or renal) adj2 (disease$ or disorder$ or insufficien$ or fail$)) or ((kidney or renal) adj2 (transplant$ or graft$ or allograft$))).ti. or (CKF or CKD or CRF or CRD or ESRF or ESKF or ESKD or ESRD or CAPD or CCPD or APD).ti,ab. or (renal replacement$ or hemodialysis or haemodialysis or hemofiltration or haemofiltration or hemodiafiltration or haemodiafiltration or predialysis or pre-dialysis or dialysis).ti.
7	((solid organ or kidney or heart or liver or intestin$ or lung or pancreas or hematopoietic stem cell$ or blood stem cell$ or bone marrow) adj5 transplant$).ti.
8	(primary immunodeficienc$ or primary immune deficienc$ or diGeorge or wiskott-aldrich or PID or PIDDs).ti.
9	chronic liver disease.ti.
10	cirrhosis.ti.
11	(NASH or ((nonalcoholic or non-alcoholic) adj3 (steatohepatitis or fatty liver))).ti.
12	(alcoholic liver disease$ or alcoholic liver disorder$).ti.
13	(autoimmune hepatitis or lupoid hepatitis).ti.
14	(chronic airflow obstruction$ or chronic airway obstruction$ or chronic obstructive bronchopulmonary disease$ or chronic obstructive lung disorder$ or chronic obstructive pulmonary disease$ or chronic obstructive pulmonary disorder$ or chronic obstructive respiratory disease$ or chronic pulmonary obstructive disease$ or chronic pulmonary obstructive disorder$ or copd$ or lung chronic obstructive disease$ or obstructive chronic lung disease$ or obstructive chronic pulmonary disease$).ti.

15	(cystic fibrosis or cystic pancreas fibrosis or cystic pancreatic fibrosis or fibrocystic disease or mckusick 21970 or mucoviscidos?s or pancreas cystic disease or pancreas cystic fibrosis or pancreas fibrocystic disease or pancreas fibrosis or pancreatic cystic disease or pancreatic cystic fibrosis or pancreatic fibrosis).ti.
16	(type 1 diabet$ or type I diabet$ or t1dm or insulin-dependent diabet$ or IDDM).ti.
17	(type 2 diabet$ or type II diabet$ or t2dm or non-insulin-dependent diabet$ or noninsulin-dependent diabet$ or NIDDM).ti.
18	(down syndrome$ or trisomy 13$).ti.
19	(Disabilit$ or disabled or (impair* adj2 (physical* or visual$ or vision$ or hear$ or sensory$)) or blind or deaf or handicap$ or cerebral palsy or autism or autistic or asperger$ or ADHD or Trisomy or Fragile X or Muscular Dystroph$ or Tourette$ or Epilep$ or seizure$ or Neuropath$ or neuromuscular or myasthenia$).ti.
20	(cardiac backward failure or cardiac decompensation or cardiac failure or cardiac incompetence or cardiac insufficienc$ or cardiac stand still or cardial decompensation or cardial insufficienc$ or chronic heart insufficienc$ or decompensatio cordis or heart backward failure or heart decompensation or heart failure or heart incompetence or heart insufficienc$ or insufficientia cardis or myocardial failure or myocardial insufficienc$ or CHF or HFREF or HFPEF).ti.
21	(acute coronary syndrome$ or acs or Myocardial Infarction$ or AMI or STEMI or NSTEMI or non-stemi or Unstable Angina or Myocardial preinfarct$ or Myocardial pre-infarct$ or Preinfarct angina or pre-infarct angina or preinfarction angina or pre-infarction angina or Angina at rest or Variant angina or Prinzmetals angina or cardiac allograft vasculopathy or (coronary artery adj2 (aneurysm or anomaly or atherosclerosis or calcification or constriction or dissection or obstruction or occlusion or perforation or thrombosis)) or coronary bifurcation lesion or coronary subclavian steal syndrome or kounis syndrome or no reflow phenomenon).ti.
22	(cardiomyopathy or barth syndrome or chagas or heart amyloidosis or heart right ventricle dysplasia or kearns sayre syndrome or ventricular noncompaction).ti.
23	(HIV or human immunodeficiency virus).ti.
24	(diffuse interstitial pneumopath$ or diffuse parenchyma lung disease$ or diffuse parenchymal lung disease or diffuse parenchymal pulmonary disease$ or diffuse parenchymal pulmonary disorder or interstitial lung disease$ or interstitial lung disorder$ or interstitial pneumopathy or interstitial pulmonary disease or interstitial pulmonary disorder or antisynthetase syndrome or berylliosis or fibrosing alveolitis or interstitial pneumonia or loeffler pneumonia or pneumoconiosis or wegener granulomatosis).ti.
25	(((mood or affective) adj2 (disorder$ or disturbance or illness)) or affective neurosis or affective psychosis or blunted affect or depression$ or depressive or bipolar or dysphoria or dysthymia or melancholia or perry syndrome or premenstrual dysphoric disorder or pseudodementia or seasonal affective disorder or major affective disorder or mania or hypomania or manic$ or minor affective disorder or schizoaffective psychosis).ti.
26	(schizophrenia or schizophrenic or dementia praecox).ti.
27	(neurocognitive disorder$ or cognitive impairment$ or dementia$ or amentia$).ti.
28	(obesity or adipose tissue hyperplasia or adipositas or adiposity or excess body weight or corpulency or fat overload syndrome or obesitas or overweight or lipedema or metabolic syndrome).ti.
29	physical inactivit$.ti.
30	(pregnan$ or gestation or gravidity or child-bearing or childbearing or post-partum or postpartum or puerperium or puerperal or weaning).ti.
31	(pulmonary arterial hypertension or pulmonary artery hypertension or lung artery hypertension or lung arterial hypertension or lung hypertension or pulmonary hypertensive disease or pulmonary hypertensive disorder or pulmonary hypertension or pulmonary embol$ or lung embol$ or lung microembol$ or pulmonary thromboembol$).ti.

32	(smoking or smoker$ or "tobacco use" or non-smoker$ or ex-smoker$ or never-smoker$ or nonsmoker$).ti.
33	(tuberculosis or tuberculous).ti.
34	(steroid$ or corticosteroid$ or abiraterone acetate or androstane derivative or azasteroid or catatoxic steroid or chandonium iodide or clascoterone or diosgenin or estrane derivative or etiocholanolone or fluasterone or ganaxolone or gonane derivative or hecogenin or homosteroid or hydroxysteroid or mipicoledine or neurosteroid or norsteroid or oxosteroid or pamaqueside or pregnane derivative or rocuronium or rostafuroxin or sarsasapogenin or secosteroid or smilagenin or sonolisib or spirostan derivative or brassinolide or nassinosteroid or casasterone or ecdysone or ecdysteroid or ecdysterone or ponasterone A or hydroxycorticosteroid or henzodrocortisone or glucocorticoid or alclometasone or algestone or amcinonide or amelometasone or beclometasone or betamethasone or celestamine or budesonide or butixocort or (chlorhexidine acetate and tixocortol pivalate) or chloroprednisone or (chlorpheniramine maleate and cortisone acetate) or (chlorquinaldol and promestriene) or ciclesonide or ciprocinonide or (ciprofloxacin and fluocinolone acetonide) or (clioquinol and flumetasone pivalate) or clobetasol or clobetasone or clocortolone or cloprednol or cortisone or tetrahydrocortisone or cortivazol or deflazacort or dexamethasone or baycuter or desoximetasone or dexatopic or sofradex or diflorasone or diflucortolone or difluprednate or domoprednate or drocinonide or dutimelan or etiprednol dicloacetate or fluclorolone or fludrocortisone or fludroxycortide or flumetasone or flumoxonide or flunisolide or fluocinolone or fluocinonide or fluocortin or fluocortolone or fluorometholone or fluprednidene or fluprednisolone or fluticasone or formocortal or halcinonide or halometasone or halopredone or hydrocortisone or epihydrocortisone or hydrasllostane or hydrocortamate or tetrahydrocortisol or icometasone enbutate or isoflupredone or itrocinonide or locicortolone dicibate or lorinden-a or lorinden-t or loteprednol or mazipredone or medrysone or meprednisone or mometasone furoate or nicocortonide or nivacortol or oropivalone or paramethasone or prednisolone or prednisone or pregnenolone or procinonide or promestriene or resocortol or rimexolone or rofleponide or ticabesone or timobesone or tipredane or tixocortol or triamcinolone or mycolog or ulobetasol propionate or uniderm or vamorolone or zoticasone or mineralocorticoid or aldosterone or corticosterone or dehydrocorticosterone or deoxycorticosterone or tetrahydroeoxycorticosterone or fludrocortisone or vecuronium or zuranolone).ti.
35	(immunosuppress$ or abatacept or abetimus or aldophosphamide or alemtuzumab or amsacrine or anifrolumab or anisperimus or apilimod or ascomycin or ascrolimus or aselizumab or atacicept or atorolimumab or avacopan or avizakimab or azathioprine or basiliximab or batoclimab or batriden or beclomethasone dipropionate or begelomab or belatacept or belimumab or betamethasone dipropionate or blisibimod or brepocitinib or briobacept or carisoprodol plus prednisolone or cedelizumab or cendakimab or cenerimod or cenplacel-L or certolizumab pegol or cm-4620 or CM4620 or colchicine or cusatuzumab or cyclophosphamide or cyclosporin$ or daclizumab or dafsolimab setaritox or darvadstrocel or daxdilimab or dazodalibep or deflazacort or defoslimod or dehydrodidemnin B or dexamethasone or didemnin-A or didemnin-B or dihydrocyclosporin A or dimethyl fumarate or dorlimomab aritox or ebdarokimab or ect-001 or edratide or efalizumab or eldelumab or elsilimomab or elsubrutinib or emapalumab or enlimomab or enlimomab pegol or erlizumab or etrasimod or faralimomab or fezakinumab or filgotinib or fingolimod or fontolizumab or forigerimod or forodesine or fosifidancitinib or fr-900523 or fr900523 or fr-900525 or fr900525 or glatiramer or govitecan or grisnilimab setaritox or guselkumab or ifidancitinib or imilecleucel-t or imlifidase or inebilizumab or inolimomab or interleukin-2 receptor antibody or irinotecan or izencitinib or laflunimus or lazucirnon or lebrikizumab or leflunomide or lerociclib or letolizumab or levilimab or lirentelimab or ljp-1082 or lusvertikimab or malononitrilamide or manitimus or maslimomab or merimepodib or methotrexate or mizoribine or mocravimod or morolimumab or mycophenolate mofetil or mycophenolic acid or napirimus or narsoplimab or natalizumab or nimacimab or nipocalimab or obexelimab or ocrelizumab or ofatumumab or OKT-3 or olamkicept or olcorolimus or olendalizumab or omalizumab or orazipone or oxeclosporin or ozanimod or paquinimod or pascolizumab or pateclizumab or peldesine or perfosfamide or pimecrolimus or plovamer or pn-1007 or Polypodium leucotomos extract or ponesimod or pritoxaximab or prodigiosin or ramatercept or rapamycin or rb-212 or rb212 or relfovetmab or remestemcel-L or reprimun or reproxalap or rilonacept or rilzabrutinib or rimiducid or risankizumab or rlyb211 or rozanolixizumab or ruclosporin or sanglifehrin-A or sonelokimab or sotrastaurin or sufosfamide or suppressor factor or tacrolimus or telimomab aritox or temsirolimus or teneliximab or teplizumab or thermozymocidin or tiplimotide or tocilizumab or tofacitinib or tol2 or traxanox or tresperimus or

	trilaciclib or tumor necrosis factor inhibitor$ or TNF-inhibitor$ or adalimumab or amlitelimab or belantamab or bleselumab or CD24Fc or cudarolimab or denosumab or efizonerimod alfa or etanercept or giloralimab or golimumab or infliximab or mitazalimab or pavurutamab or pegilodecakin or peimine or quellor or ravagalimab or remtolumab or selicrelumab or sibeprenlimab or sotigalimab or tanfanercept or tavolimab or telazorlimab or telitacicept or tibulizumab or umirolimus or valziflocept or vapaliximab or vepalimomab or vgx-1027 or vgx1027 or vidofludimus or voclosporin or zolimomab aritox or zotarolimus).ti.
36	or/2-35
37	1 and 36
38	exp Hospitalization/ or intensive care unit/ or intensive care/ or exp Artificial Respiration/ or exp absenteeism/ or (healthcare resource or healthcare resources or medical resource or medical resources or health resource consumption or health care consumption or 'healthcare resource use' or medical resource consumption or hospitali?ation or hospital admission or hospital admissions or icu admission or icu admissions or emergency department visit or emergency department visits or emergency room visit or emergency room visits or er visit or er visits or ed visit or ed visits or inpatient visit or inpatient visits or outpatient visit or outpatient visits or specialist visit or specialist visits or unscheduled doctor visit or unscheduled doctor visits or unscheduled physician visit$ or general practitioner visit$ or mechanical ventilation$ or non-invasive ventilation$ or noninvasive ventilation$ or CPAP$ or Continuous Positive Airway Pressure$ or Airway Pressure Release Ventilation or APRV or BiPAP or length of stay or LOS or absenteeism or presenteeism or work product$ or WPAI$ or productivity loss or economic burden).ti,ab.
39	exp quality of life/ or (qol or quality of life or hrql or hrqol or quality adjusted life year$ or qaly or patient reported outcome$ or satisfaction or preference$ or activities of daily living or adl or assessment of quality of life or aqol or quality of well being scale or mental health or anxiety or anxious or depress$ or psycholog$).ti,ab.
40	((utilit* and health) or (utilit* and scor*) or (utilit* and valu*) or (disutilit* and health) or (disutilit* and scor*) or (disutilit* and valu*) or daly or dalys or disability adjusted life year$ or standard gamble or time trade-off or time tradeoff or visual analog$ scale or discrete choice experiment or qwb or 15d or health utilities index or hui or hui1 or hui2 or hui3).ti,ab.
41	(sf36 or sf-36 or sf6 or sf-6 or short form 6 or sf6d or sf-6d or short form 6d or eq-5d or eq5d or euroqol or euro-qol or health status or hye or hyes or health$ year$ equivalent$ or rosser index or quality of wellbeing or qwb).ti,ab.
42	exp Health Care Costs/ or exp Drug Costs/ or exp "Cost of Illness"/ or exp Hospital Costs/ or exp Economics, Pharmaceutical/ or (treatment cost$ or direct cost$ or direct medical cost$ or nonmedical cost$ or non-medical cost$ or total cost or total costs or cost per patient treated or budget impact or cost burden or societal cost$ or administrative cost$ or travel cost$ or travel time or disease cost or cost of drugs).ti,ab.
43	exp isolation/ or (shield$ or isolat$).ti,ab.
44	loneliness/ or (lonely or loneliness).ti,ab.
45	social distancing/ or ((social or physical) adj distanc$).ti,ab.
46	or/38-45
47	37 and 46
48	exp longitudinal study/ or exp retrospective study/ or exp prospective study/ or exp cohort analysis/ or exp cross-sectional study/ or exp cohort analysis/ or exp observational study/ or (longitudinal study or retrospective study or prospective study or cohort$ or follow up or cross-sectional study or cross sectional study or followup study or observational study or registry or registries or real world or cross sectional or RWE).ti,ab.
49	47 and 48
50	exp book/ or exp theoretical study/ or exp case report/ or (letter or editorial or erratum or note or short survey).pt.

51	case study/ or case report$.jx. or case report$.jw. or (case report or case series or woman or man or child or adolescent or female or male or boy or girl or infant).ti.
52	review.pt. not (systematic or (meta and analy$) or ((indirect or mixed) and treatment comparison)).ti,ab.
53	(exp animal/ or nonhuman/) not exp human/
54	(Ephemera or "Introductory Journal Article" or News or "Newspaper Article" or Editorial or Comment or Overall).pt. or in vitro study/ or (commentary or editorial or comment or letter or mice or rat or mouse or animal or murine).ti.
55	exp clinical trial/ or exp randomized controlled trial/
56	or/50-55
57	49 not 56
58	limit 57 to (article or article in press or "preprints (unpublished, non-peer reviewed)")
59	limit 58 to yr="2021-Current"

Supplemental Table 2. Medline search strategy.

No.	Search Terms
1	(covid-19 or covid19 or corona-virus or sars-cov-2 or sars-cov2 or coronavirus disease or ncov or n-cov or delta or omicron).ti.
2	asthma$.ti.
3	(bronchiectasis or bronchiectasia or bronchoectasia).ti.
4	(cancer or neoplasm$ or leuk?emia or lymphoma or mesothelioma or myeloma or melanoma or carcinoma or adenocarcinoma or sarcoma or hemangioma or schwannoma$ or teratoma$ or tumo?r or tumo?rs).ti.
5	(brain angiopathy or brain circulation failure or brain vascular disease or brain vasculopathy or cerebral small vessel disease$ or cerebral vascular disease or cerebral vascular disorder or cerebral vascular disturbance or cerebral vascular lesion or cerebral vasculopathy or cerebrovascular damage or cerebrovascular disorder$ or cerebrovascular lesion or cerebrovascular pathology or cerebrovascular syndrome or basal ganglion h?emorrhage or bow hunter syndrome$ or brain hemangioma or brain hematoma or ((((brain or cerebral or corpus callosum or intracranial or intracerebral or periventricular or posterior fossa) adj2 (h?emorrhage or h?emorrhagic)) or ((brain or cerebral or cerebrovascular or hemisphere) adj2 infarct$) or ((brain or cerebral or cerebrovascular or cerebri or neural) adj2 (isch?emia or arterial insufficiency or circulation disorder$ or blood flow disorder or circulation disorder or circulatory disorder or encephalopathy)) or brain vasospasm or ischemic encephalopathy or transient ischemic attack or carotid artery disease$ or carotid arterial disease or carotid arteriopathy or carotid disease or (carotid artery adj2 (aneurysm or anomaly or bruit or calcification or injury or obstruction or atherosclerosis)) or moyamoya disease or cerebral artery disease$ or cerebrovascular accident or stroke or apoplexia or apoplexy or ((brain or cerebral or cerebrovascular) adj2 (accident or attack or insult or apoplexia or failure)) or cerebrovascular malformation or brain arteriovenous malformation or vein of galen malformation or hypophysis apoplexy or intracranial aneurysm or (brain or cerebral or cerebrovascular or intracranial)) adj2 (obstruction or occlusion or thrombosis or phlebothrombosis or thromboembolism or thrombosis or occlusive)) or melas syndrome or occlusive cerebrovascular disease or ocular isch?emic syndrome or posterior reversible encephalopathy syndrome or vertebrobasilar insufficiency or Wallenberg syndrome).ti.
6	(((chronic kidney or renal) adj2 (disease$ or disorder$ or insufficien$ or fail$)) or ((kidney or renal) adj2 (transplant$ or graft$ or allograft$))).ti. or (CKF or CKD or CRF or CRD or ESRF or ESKF or ESKD or ESRD or CAPD or CCPD or APD).ti,ab. or (renal replacement$ or hemodialysis or haemodialysis or hemofiltration or haemofiltration or hemodiafiltration or haemodiafiltration or predialysis or pre-dialysis or dialysis).ti.
7	((solid organ or kidney or heart or liver or intestin$ or lung or pancreas or hematopoietic stem cell$ or blood stem cell$ or bone marrow) adj5 transplant$).ti.
8	(primary immunodeficienc$ or primary immune deficienc$ or diGeorge or wiskott-aldrich or PID or PIDDs).ti.
9	chronic liver disease.ti.
10	cirrhosis.ti.
11	(NASH or ((nonalcoholic or non-alcoholic) adj3 (steatohepatitis or fatty liver))).ti.
12	(alcoholic liver disease$ or alcoholic liver disorder$).ti.
13	(autoimmune hepatitis or lupoid hepatitis).ti.
14	(chronic airflow obstruction$ or chronic airway obstruction$ or chronic obstructive bronchopulmonary disease$ or chronic obstructive lung disorder$ or chronic obstructive pulmonary disease$ or chronic obstructive pulmonary disorder$ or chronic obstructive respiratory disease$ or chronic pulmonary obstructive disease$ or chronic pulmonary obstructive disorder$ or copd$ or lung chronic obstructive disease$ or obstructive chronic lung disease$ or obstructive chronic pulmonary disease$).ti.
15	(cystic fibrosis or cystic pancreas fibrosis or cystic pancreatic fibrosis or fibrocystic disease or mckusick 21970 or mucoviscidos?s or pancreas cystic disease or pancreas cystic fibrosis or pancreas fibrocystic disease or pancreas fibrosis or pancreatic cystic disease or pancreatic cystic fibrosis or pancreatic fibrosis).ti.
16	(type 1 diabet$ or type I diabet$ or t1dm or insulin-dependent diabet$ or IDDM).ti.
17	(type 2 diabet$ or type II diabet$ or t2dm or non-insulin-dependent diabet$ or noninsulin-dependent diabet$ or NIDDM).ti.
18	(down syndrome$ or trisomy 13$).ti.
19	(Disabilit$ or disabled or (impair* adj2 (physical* or visual$ or vision$ or hear$ or sensory$)) or blind or deaf or handicap$ or cerebral palsy or autism or autistic or asperger$ or ADHD or

	Trisomy or Fragile X or Muscular Dystroph$ or Tourette$ or Epilep$ or seizure$ or Neuropath$ or neuromuscular or myasthenia$).ti.
20	(cardiac backward failure or cardiac decompensation or cardiac failure or cardiac incompetence or cardiac insufficienc$ or cardiac stand still or cardial decompensation or cardial insufficienc$ or chronic heart insufficienc$ or decompensation cordis or heart backward failure or heart decompensation or heart failure or heart incompetence or heart insufficienc$ or insufficientia cardis or myocardial failure or myocardial insufficienc$ or CHF or HFREF or HFPEF).ti.
21	(acute coronary syndrome$ or acs or Myocardial Infarction$ or AMI or STEMI or NSTEMI or non-stemi or Unstable Angina or Myocardial preinfarct$ or Myocardial pre-infarct$ or Preinfarct angina or pre-infarct angina or preinfarction angina or pre-infarction angina or Angina at rest or Variant angina or Prinzmetals angina or cardiac allograft vasculopathy or (coronary artery adj2 (aneurysm or anomaly or atherosclerosis or calcification or constriction or dissection or obstruction or occlusion or perforation or thrombosis)) or coronary bifurcation lesion or coronary subclavian steal syndrome or kounis syndrome or no reflow phenomenon).ti.
22	(cardiomyopathy or barth syndrome or chagas or heart amyloidosis or heart right ventricle dysplasia or kearns sayre syndrome or ventricular noncompaction).ti.
23	(HIV or human immunodeficiency virus).ti.
24	(diffuse interstitial pneumopath$ or diffuse parenchyma lung disease$ or diffuse parenchymal lung disease or diffuse parenchymal pulmonary disease$ or diffuse parenchymal pulmonary disorder or interstitial lung disease$ or interstitial lung disorder$ or interstitial pneumopathy or interstitial pulmonary disease or interstitial pulmonary disorder or antisynthetase syndrome or berylliosis or fibrosing alveolitis or interstitial pneumonia or loeffler pneumonia or pneumoconiosis or wegener granulomatosis).ti.
25	(((mood or affective) adj2 (disorder$ or disturbance or illness)) or affective neurosis or affective psychosis or blunted affect or depression$ or depressive or bipolar or dysphoria or dysthymia or melancholia or perry syndrome or premenstrual dysphoric disorder or pseudodementia or seasonal affective disorder or major affective disorder or mania or hypomania or manic$ or minor affective disorder or schizoaffective psychosis).ti.
26	(schizophrenia or schizophrenic or dementia praecox).ti.
27	(neurocognitive disorder$ or cognitive impairment$ or dementia$ or amentia$).ti.
28	(obesity or adipose tissue hyperplasia or adipositas or adiposity or excess body weight or corpulency or fat overload syndrome or obesitas or overweight or lipedema or metabolic syndrome).ti.
29	physical inactivit$.ti.
30	(pregnan$ or gestation or gravidity or child-bearing or childbearing or post-partum or postpartum or puerperium or puerperal or weaning).ti.
31	(pulmonary arterial hypertension or pulmonary artery hypertension or lung artery hypertension or lung arterial hypertension or lung hypertension or pulmonary hypertensive disease or pulmonary hypertensive disorder or pulmonary hypertension or pulmonary embol$ or lung embol$ or lung microembol$ or pulmonary thromboembol$).ti.
32	(smoking or smoker$ or "tobacco use" or non-smoker$ or ex-smoker$ or never-smoker$ or nonsmoker$).ti.
33	(tuberculosis or tuberculous).ti.
34	(steroid$ or corticosteroid$ or abiraterone acetate or androstane derivative or azasteroid or catatoxic steroid or chandonium iodide or clascoterone or diosgenin or estrane derivative or etiocholanolone or fluasterone or ganaxolone or gonane derivative or hecogenin or homosteroid or hydroxysteroid or mipicoledine or neurosteroid or norsteroid or oxosteroid or pamaqueside or pregnane derivative or rocuronium or rostafuroxin or sarsasapogenin or secosteroid or smilagenin or sonolisib or spirostan derivative or brassinolide or nassinosteroid or casasterone or ecdysone or ecdysteroid or ecdysterone or ponasterone A or hydroxycorticosteroid or henzodrocortisone or glucocorticoid or alclometasone or algestone or amcinonide or amelometasone or beclometasone or betamethasone or celestamine or budesonide or butixocort or (chlorhexidine acetate and tixocortol pivalate) or chloroprednisone or (chlorpheniramine maleate and cortisone acetate) or (chlorquinaldol and promestriene) or ciclesonide or ciprocinonide or (ciprofloxacin and fluocinolone acetonide) or (clioquinol and flumetasone pivalate) or clobetasol or clobetasone or clocortolone or cloprednol or cortisone or tetrahydrocortisone or cortivazol or deflazacort or dexamethasone or baycuter or desoximetasone or dexatopic or sofradex or diflorasone or diflucortolone or difluprednate or domoprednate or drocinonide or dutimelan or etiprednol dicloacetate or fluclorolone or fludrocortisone or fludroxycortide or flumetasone or flumoxonide or flunisolide or fluocinolone or fluocinonide or fluocortin or fluocortolone or fluorometholone or fluprednidene or fluprednisolone or fluticasone or formocortal or halcinonide or halometasone or

	halopredone or hydrocortisone or epihydrocortisone or hydrasllostane or hydrocortamate or tetrahydrocortisol or icometasone enbutate or isoflupredone or itrocinonide or locicortolone dicibate or lorinden-a or lorinden-t or loteprednol or mazipredone or medrysone or meprednisone or mometasone furoate or nicocortonide or nivacortol or oropivalone or paramethasone or prednisolone or prednisone or pregnenolone or procinonide or promestriene or resocortol or rimexolone or rofleponide or ticabesone or timobesone or tipredane or tixocortol or triamcinolone or mycolog or ulobetasol propionate or uniderm or vamorolone or zoticasone or mineralocorticoid or aldosterone or corticosterone or dehydrocorticosterone or deoxycorticosterone or tetrahydroeoxycorticosterone or fludrocortisone or vecuronium or zuranolone).ti.
35	(immunosuppress$ or abatacept or abetimus or aldophosphamide or alemtuzumab or amsacrine or anifrolumab or anisperimus or apilimod or ascomycin or ascrolimus or aselizumab or atacicept or atorolimumab or avacopan or avizakimab or azathioprine or basiliximab or batoclimab or batriden or beclomethasone dipropionate or begelomab or belatacept or belimumab or betamethasone dipropionate or blisibimod or brepocitinib or briobacept or carisoprodol plus prednisolone or cedelizumab or cendakimab or cenerimod or cenplacel-L or certolizumab pegol or cm-4620 or CM4620 or colchicine or cusatuzumab or cyclophosphamide or cyclosporin$ or daclizumab or dafsolimab setaritox or darvadstrocel or daxdilimab or dazodalibep or deflazacort or defoslimod or dehydrodidemnin B or dexamethasone or didemnin-A or didemnin-B or dihydrocyclosporin A or dimethyl fumarate or dorlimomab aritox or ebdarokimab or ect-001 or edratide or efalizumab or eldelumab or elsilimomab or elsubrutinib or emapalumab or enlimomab or enlimomab pegol or erlizumab or etrasimod or faralimomab or fezakinumab or filgotinib or fingolimod or fontolizumab or forigerimod or forodesine or fosifidancitinib or fr-900523 or fr900523 or fr-900525 or fr900525 or glatiramer or govitecan or grisnilimab setaritox or guselkumab or ifidancitinib or imilecleucel-t or imlifidase or inebilizumab or inolimomab or interleukin-2 receptor antibody or irinotecan or izencitinib or laflunimus or lazucirnon or lebrikizumab or leflunomide or lerociclib or letolizumab or levilimab or lirentelimab or ljp-1082 or lusvertikimab or malononitrilamide or manitimus or maslimomab or merimepodib or methotrexate or mizoribine or mocravimod or morolimumab or mycophenolate mofetil or mycophenolic acid or napirimus or narsoplimab or natalizumab or nimacimab or nipocalimab or obexelimab or ocrelizumab or ofatumumab or OKT-3 or olamkicept or olcorolimus or olendalizumab or omalizumab or orazipone or oxeclosporin or ozanimod or paquinimod or pascolizumab or pateclizumab or peldesine or perfosfamide or pimecrolimus or plovamer or pn-1007 or Polypodium leucotomos extract or ponesimod or pritoxaximab or prodigiosin or ramatercept or rapamycin or rb-212 or rb212 or relfovetmab or remestemcel-L or reprimun or reproxalap or rilonacept or rilzabrutinib or rimiducid or risankizumab or rlyb211 or rozanolixizumab or ruclosporin or sanglifehrin-A or sonelokimab or sotrastaurin or sufosfamide or suppressor factor or tacrolimus or telimomab aritox or temsirolimus or teneliximab or teplizumab or thermozymocidin or tiplimotide or tocilizumab or tofacitinib or tol2 or traxanox or tresperimus or trilaciclib or tumor necrosis factor inhibitor$ or TNF-inhibitor$ or adalimumab or amlitelimab or belantamab or bleselumab or CD24Fc or cudarolimab or denosumab or efizonerimod alfa or etanercept or giloralimab or golimumab or infliximab or mitazalimab or pavurutamab or pegilodecakin or peimine or quellor or ravagalimab or remtolumab or selicrelumab or sibeprenlimab or sotigalimab or tanfanercept or tavolimab or telazorlimab or telitacicept or tibulizumab or umirolimus or valziflocept or vapaliximab or vepalimomab or vgx-1027 or vgx1027 or vidofludimus or voclosporin or zolimomab aritox or zotarolimus).ti.
36	or/2-35
37	1 and 36
38	exp Hospitalization/ or intensive care unit/ or intensive care/ or exp Artificial Respiration/ or exp absenteeism/ or (healthcare resource or healthcare resources or medical resource or medical resources or health resource consumption or health care consumption or 'healthcare resource use' or medical resource consumption or hospitali?ation or hospital admission or hospital admissions or icu admission or icu admissions or emergency department visit or emergency department visits or emergency room visit or emergency room visits or er visit or er visits or ed visit or ed visits or inpatient visit or inpatient visits or outpatient visit or outpatient visits or specialist visit or specialist visits or unscheduled doctor visit or unscheduled doctor visits or unscheduled physician visit$ or general practitioner visit$ or mechanical ventilation$ or non-invasive ventilation$ or noninvasive ventilation$ or CPAP$ or Continuous Positive Airway Pressure$ or Airway Pressure Release Ventilation or APRV or BiPAP or length of stay or LOS or absenteeism or presenteeism or work product$ or WPAI$ or productivity loss or economic burden).ti,ab.
39	exp quality of life/ or (qol or quality of life or hrql or hrqol or quality adjusted life year$ or qaly or patient reported outcome$ or satisfaction or preference$ or activities of daily living or adl or

	assessment of quality of life or aqol or quality of well being scale or mental health or anxiety or anxious or depress$ or psycholog$).ti,ab.
40	((utilit* and health) or (utilit* and scor*) or (utilit* and valu*) or (disutilit* and health) or (disutilit* and scor*) or (disutilit* and valu*) or daly or dalys or disability adjusted life year$ or standard gamble or time trade-off or time tradeoff or visual analog$ scale or discrete choice experiment or qwb or 15d or health utilities index or hui or hui1 or hui2 or hui3).ti,ab.
41	(sf36 or sf-36 or sf6 or sf-6 or short form 6 or sf6d or sf-6d or short form 6d or eq-5d or eq5d or euroqol or euro-qol or health status or hye or hyes or health$ year$ equivalent$ or rosser index or quality of wellbeing or qwb).ti,ab.
42	exp Health Care Costs/ or exp Drug Costs/ or exp "Cost of Illness"/ or exp Hospital Costs/ or exp Economics, Pharmaceutical/ or (treatment cost$ or direct cost$ or direct medical cost$ or nonmedical cost$ or non-medical cost$ or total cost or total costs or cost per patient treated or budget impact or cost burden or societal cost$ or administrative cost$ or travel cost$ or travel time or disease cost or cost of drugs).ti,ab.
43	exp patient isolation/ or (shield$ or isolat$).ti,ab.
44	loneliness/ or (lonely or loneliness).ti,ab.
45	physical distancing/ or ((social or physical) adj distanc$).ti,ab.
46	or/38-45
47	37 and 46
48	exp Longitudinal Studies/ or exp Retrospective Studies/ or exp Prospective Studies/ or exp Cohort Studies/ or exp Cross-Sectional Studies/ or (longitudinal study or retrospective study or prospective study or cohort$ or follow up or cross-sectional study or cross sectional study or followup study or observational study or registry or registries or real world or cross sectional or RWE).ti,ab.
49	47 and 48
50	exp book/ or exp theoretical study/ or exp case report/ or (letter or editorial or erratum or note or short survey).pt.
51	case reports.pt. or case report$.jw. or (case report or case series or woman or man or child or adolescent or female or male or boy or girl or infant).ti.
52	review.pt. not (systematic or (meta and analy$) or ((indirect or mixed) and treatment comparison)).ti,ab.
53	exp animals/ not exp humans/
54	(Ephemera or "Introductory Journal Article" or News or "Newspaper Article" or Editorial or Comment or Overall).pt. or in vitro study/ or (commentary or editorial or comment or letter or mice or rat or mouse or animal or murine).ti.
55	exp clinical trial/ or exp randomized controlled trial/
56	or/50-55
57	49 not 56
58	limit 57 to yr="2021-Current"

Supplemental Table 3. PsycInfo search strategy.

No.	Search Terms
1	(covid-19 or covid19 or corona-virus or sars-cov-2 or sars-cov2 or coronavirus disease or ncov or n-cov or delta or omicron).ti.
2	asthma$.ti.
3	(bronchiectasis or bronchiectasia or bronchoectasia).ti.
4	(cancer or neoplasm$ or leuk?emia or lymphoma or mesothelioma or myeloma or melanoma or carcinoma or adenocarcinoma or sarcoma or hemangioma or schwannoma$ or teratoma$ or tumo?r or tumo?rs).ti.
5	(brain angiopathy or brain circulation failure or brain vascular disease or brain vasculopathy or cerebral small vessel disease$ or cerebral vascular disease or cerebral vascular disorder or cerebral vascular disturbance or cerebral vascular lesion or cerebral vasculopathy or cerebrovascular damage or cerebrovascular disorder$ or cerebrovascular lesion or cerebrovascular pathology or cerebrovascular syndrome or basal ganglion h?emorrhage or bow hunter syndrome$ or brain hemangioma or brain hematoma or ((((brain or cerebral or corpus callosum or intracranial or intracerebral or periventricular or posterior fossa) adj2 (h?emorrhage or h?emorrhagic)) or ((brain or cerebral or cerebrovascular or hemisphere) adj2 infarct$) or ((brain or cerebral or cerebrovascular or cerebri or neural) adj2 (isch?emia or arterial insufficiency or circulation disorder$ or blood flow disorder or circulation disorder or circulatory disorder or encephalopathy)) or brain vasospasm or ischemic encephalopathy or transient ischemic attack or carotid artery disease$ or carotid arterial disease or carotid arteriopathy or carotid disease or (carotid artery adj2 (aneurysm or anomaly or bruit or calcification or injury or obstruction or atherosclerosis)) or moyamoya disease or cerebral artery disease$ or cerebrovascular accident or stroke or apoplexia or apoplexy or ((brain or cerebral or cerebrovascular) adj2 (accident or attack or insult or apoplexia or failure)) or cerebrovascular malformation or brain arteriovenous malformation or vein of galen malformation or hypophysis apoplexy or intracranial aneurysm or (brain or cerebral or cerebrovascular or intracranial)) adj2 (obstruction or occlusion or thrombosis or phlebothrombosis or thromboembolism or thrombosis or occlusive)) or melas syndrome or occlusive cerebrovascular disease or ocular isch?emic syndrome or posterior reversible encephalopathy syndrome or vertebrobasilar insufficiency or Wallenberg syndrome).ti.
6	(((chronic kidney or renal) adj2 (disease$ or disorder$ or insufficien$ or fail$)) or ((kidney or renal) adj2 (transplant$ or graft$ or allograft$))).ti. or (CKF or CKD or CRF or CRD or ESRF or ESKF or ESKD or ESRD or CAPD or CCPD or APD).ti,ab. or (renal replacement$ or hemodialysis or haemodialysis or hemofiltration or haemofiltration or hemodiafiltration or haemodiafiltration or predialysis or pre-dialysis or dialysis).ti.
7	((solid organ or kidney or heart or liver or intestin$ or lung or pancreas or hematopoietic stem cell$ or blood stem cell$ or bone marrow) adj5 transplant$).ti.
8	(primary immunodeficienc$ or primary immune deficienc$ or diGeorge or wiskott-aldrich or PID or PIDDs).ti.
9	chronic liver disease.ti.
10	cirrhosis.ti.
11	(NASH or ((nonalcoholic or non-alcoholic) adj3 (steatohepatitis or fatty liver))).ti.
12	(alcoholic liver disease$ or alcoholic liver disorder$).ti.
13	(autoimmune hepatitis or lupoid hepatitis).ti.
14	(chronic airflow obstruction$ or chronic airway obstruction$ or chronic obstructive bronchopulmonary disease$ or chronic obstructive lung disorder$ or chronic obstructive pulmonary disease$ or chronic obstructive pulmonary disorder$ or chronic obstructive respiratory disease$ or chronic pulmonary obstructive disease$ or chronic pulmonary obstructive disorder$ or copd$ or lung chronic obstructive disease$ or obstructive chronic lung disease$ or obstructive chronic pulmonary disease$).ti.
15	(cystic fibrosis or cystic pancreas fibrosis or cystic pancreatic fibrosis or fibrocystic disease or mckusick 21970 or mucoviscidos?s or pancreas cystic disease or pancreas cystic fibrosis or pancreas fibrocystic disease or pancreas fibrosis or pancreatic cystic disease or pancreatic cystic fibrosis or pancreatic fibrosis).ti.
16	(type 1 diabet$ or type I diabet$ or t1dm or insulin-dependent diabet$ or IDDM).ti.
17	(type 2 diabet$ or type II diabet$ or t2dm or non-insulin-dependent diabet$ or noninsulin-dependent diabet$ or NIDDM).ti.
18	(down syndrome$ or trisomy 13$).ti.
19	(Disabilit$ or disabled or (impair* adj2 (physical* or visual$ or vision$ or hear$ or sensory$)) or blind or deaf or handicap$ or cerebral palsy or autism or autistic or asperger$ or ADHD or

	Trisomy or Fragile X or Muscular Dystroph$ or Tourette$ or Epilep$ or seizure$ or Neuropath$ or neuromuscular or myasthenia$).ti.
20	(cardiac backward failure or cardiac decompensation or cardiac failure or cardiac incompetence or cardiac insufficienc$ or cardiac stand still or cardial decompensation or cardial insufficienc$ or chronic heart insufficienc$ or decompensatio cordis or heart backward failure or heart decompensation or heart failure or heart incompetence or heart insufficienc$ or insufficientia cardis or myocardial failure or myocardial insufficienc$ or CHF or HFREF or HFPEF).ti.
21	(acute coronary syndrome$ or acs or Myocardial Infarction$ or AMI or STEMI or NSTEMI or non-stemi or Unstable Angina or Myocardial preinfarct$ or Myocardial pre-infarct$ or Preinfarct angina or pre-infarct angina or preinfarction angina or pre-infarction angina or Angina at rest or Variant angina or Prinzmetals angina or cardiac allograft vasculopathy or (coronary artery adj2 (aneurysm or anomaly or atherosclerosis or calcification or constriction or dissection or obstruction or occlusion or perforation or thrombosis)) or coronary bifurcation lesion or coronary subclavian steal syndrome or kounis syndrome or no reflow phenomenon).ti.
22	(cardiomyopathy or barth syndrome or chagas or heart amyloidosis or heart right ventricle dysplasia or kearns sayre syndrome or ventricular noncompaction).ti.
23	(HIV or human immunodeficiency virus).ti.
24	(diffuse interstitial pneumopath$ or diffuse parenchyma lung disease$ or diffuse parenchymal lung disease or diffuse parenchymal pulmonary disease$ or diffuse parenchymal pulmonary disorder or interstitial lung disease$ or interstitial lung disorder$ or interstitial pneumopathy or interstitial pulmonary disease or interstitial pulmonary disorder or antisynthetase syndrome or berylliosis or fibrosing alveolitis or interstitial pneumonia or loeffler pneumonia or pneumoconiosis or wegener granulomatosis).ti.
25	(((mood or affective) adj2 (disorder$ or disturbance or illness)) or affective neurosis or affective psychosis or blunted affect or depression$ or depressive or bipolar or dysphoria or dysthymia or melancholia or perry syndrome or premenstrual dysphoric disorder or pseudodementia or seasonal affective disorder or major affective disorder or mania or hypomania or manic$ or minor affective disorder or schizoaffective psychosis).ti.
26	(schizophrenia or schizophrenic or dementia praecox).ti.
27	(neurocognitive disorder$ or cognitive impairment$ or dementia$ or amentia$).ti.
28	(obesity or adipose tissue hyperplasia or adipositas or adiposity or excess body weight or corpulency or fat overload syndrome or obesitas or overweight or lipedema or metabolic syndrome).ti.
29	physical inactivit$.ti.
30	(pregnan$ or gestation or gravidity or child-bearing or childbearing or post-partum or postpartum or puerperium or puerperal or weaning).ti.
31	(pulmonary arterial hypertension or pulmonary artery hypertension or lung artery hypertension or lung arterial hypertension or lung hypertension or pulmonary hypertensive disease or pulmonary hypertensive disorder or pulmonary hypertension or pulmonary embol$ or lung embol$ or lung microembol$ or pulmonary thromboembol$).ti.
32	(smoking or smoker$ or "tobacco use" or non-smoker$ or ex-smoker$ or never-smoker$ or nonsmoker$).ti.
33	(tuberculosis or tuberculous).ti.
34	(steroid$ or corticosteroid$ or abiraterone acetate or androstane derivative or azasteroid or catatoxic steroid or chandonium iodide or clascoterone or diosgenin or estrane derivative or etiocholanolone or fluasterone or ganaxolone or gonane derivative or hecogenin or homosteroid or hydroxysteroid or mipicoledine or neurosteroid or norsteroid or oxosteroid or pamaqueside or pregnane derivative or rocuronium or rostafuroxin or sarsasapogenin or secosteroid or smilagenin or sonolisib or spirostan derivative or brassinolide or nassinosteroid or casasterone or ecdysone or ecdysteroid or ecdysterone or ponasterone A or hydroxycorticosteroid or henzodrocortisone or glucocorticoid or alclometasone or algestone or amcinonide or amelometasone or beclometasone or betamethasone or celestamine or budesonide or butixocort or (chlorhexidine acetate and tixocortol pivalate) or chloroprednisone or (chlorpheniramine maleate and cortisone acetate) or (chlorquinaldol and promestriene) or ciclesonide or ciprocinonide or (ciprofloxacin and fluocinolone acetonide) or (clioquinol and flumetasone pivalate) or clobetasol or clobetasone or clocortolone or cloprednol or cortisone or tetrahydrocortisone or cortivazol or deflazacort or dexamethasone or baycuter or desoximetasone or dexatopic or sofradex or diflorasone or diflucortolone or difluprednate or domoprednate or drocinonide or dutimelan or etiprednol dicloacetate or fluclorolone or fludrocortisone or fludroxycortide or flumetasone or flumoxonide or flunisolide or fluocinolone or fluocinonide or fluocortin or fluocortolone or fluorometholone or fluprednidene or fluprednisolone or fluticasone or

	formocortal or halcinonide or halometasone or halopredone or hydrocortisone or epihydrocortisone or hydrasllostane or hydrocortamate or tetrahydrocortisol or icometasone enbutate or isoflupredone or itrocinonide or locicortolone dicibate or lorinden-a or lorinden-t or loteprednol or mazipredone or medrysone or meprednisone or mometasone furoate or nicocortonide or nivacortol or oropivalone or paramethasone or prednisolone or prednisone or pregnenolone or procinonide or promestriene or resocortol or rimexolone or rofleponide or ticabesone or timobesone or tipredane or tixocortol or triamcinolone or mycolog or ulobetasol propionate or uniderm or vamorolone or zoticasone or mineralocorticoid or aldosterone or corticosterone or dehydrocorticosterone or deoxycorticosterone or tetrahydroeoxycorticosterone or fludrocortisone or vecuronium or zuranolone).ti.
35	(immunosuppress$ or abatacept or abetimus or aldophosphamide or alemtuzumab or amsacrine or anifrolumab or anisperimus or apilimod or ascomycin or ascrolimus or aselizumab or atacicept or atorolimumab or avacopan or avizakimab or azathioprine or basiliximab or batoclimab or batriden or beclomethasone dipropionate or begelomab or belatacept or belimumab or betamethasone dipropionate or blisibimod or brepocitinib or briobacept or carisoprodol plus prednisolone or cedelizumab or cendakimab or cenerimod or cenplacel-L or certolizumab pegol or cm-4620 or CM4620 or colchicine or cusatuzumab or cyclophosphamide or cyclosporin$ or daclizumab or dafsolimab setaritox or darvadstrocel or daxdilimab or dazodalibep or deflazacort or defoslimod or dehydrodidemnin B or dexamethasone or didemnin-A or didemnin-B or dihydrocyclosporin A or dimethyl fumarate or dorlimomab aritox or ebdarokimab or ect-001 or edratide or efalizumab or eldelumab or elsilimomab or elsubrutinib or emapalumab or enlimomab or enlimomab pegol or erlizumab or etrasimod or faralimomab or fezakinumab or filgotinib or fingolimod or fontolizumab or forigerimod or forodesine or fosifidancitinib or fr-900523 or fr900523 or fr-900525 or fr900525 or glatiramer or govitecan or grisnilimab setaritox or guselkumab or ifidancitinib or imilecleucel-t or imlifidase or inebilizumab or inolimomab or interleukin-2 receptor antibody or irinotecan or izencitinib or laflunimus or lazucirnon or lebrikizumab or leflunomide or lerociclib or letolizumab or levilimab or lirentelimab or ljp-1082 or lusvertikimab or malononitrilamide or manitimus or maslimomab or merimepodib or methotrexate or mizoribine or mocravimod or morolimumab or mycophenolate mofetil or mycophenolic acid or napirimus or narsoplimab or natalizumab or nimacimab or nipocalimab or obexelimab or ocrelizumab or ofatumumab or OKT-3 or olamkicept or olcorolimus or olendalizumab or omalizumab or orazipone or oxeclosporin or ozanimod or paquinimod or pascolizumab or pateclizumab or peldesine or perfosfamide or pimecrolimus or plovamer or pn-1007 or Polypodium leucotomos extract or ponesimod or pritoxaximab or prodigiosin or ramatercept or rapamycin or rb-212 or rb212 or relfovetmab or remestemcel-L or reprimun or reproxalap or rilonacept or rilzabrutinib or rimiducid or risankizumab or rlyb211 or rozanolixizumab or ruclosporin or sanglifehrin-A or sonelokimab or sotrastaurin or sufosfamide or suppressor factor or tacrolimus or telimomab aritox or temsirolimus or teneliximab or teplizumab or thermozymocidin or tiplimotide or tocilizumab or tofacitinib or tol2 or traxanox or tresperimus or trilaciclib or tumor necrosis factor inhibitor$ or TNF-inhibitor$ or adalimumab or amlitelimab or belantamab or bleselumab or CD24Fc or cudarolimab or denosumab or efizonerimod alfa or etanercept or giloralimab or golimumab or infliximab or mitazalimab or pavurutamab or pegilodecakin or peimine or quellor or ravagalimab or remtolumab or selicrelumab or sibeprenlimab or sotigalimab or tanfanercept or tavolimab or telazorlimab or telitacicept or tibulizumab or umirolimus or valziflocept or vapaliximab or vepalimomab or vgx-1027 or vgx1027 or vidofludimus or voclosporin or zolimomab aritox or zotarolimus).ti.
36	or/2-35
37	1 and 36
38	exp Hospitalization/ or intensive care unit/ or intensive care/ or exp Artificial Respiration/ or exp absenteeism/ or (healthcare resource or healthcare resources or medical resource or medical resources or health resource consumption or health care consumption or 'healthcare resource use' or medical resource consumption or hospitali?ation or hospital admission or hospital admissions or icu admission or icu admissions or emergency department visit or emergency department visits or emergency room visit or emergency room visits or er visit or er visits or ed visit or ed visits or inpatient visit or inpatient visits or outpatient visit or outpatient visits or specialist visit or specialist visits or unscheduled doctor visit or unscheduled doctor visits or unscheduled physician visit$ or general practitioner visit$ or mechanical ventilation$ or non-invasive ventilation$ or noninvasive ventilation$ or CPAP$ or Continuous Positive Airway Pressure$ or Airway Pressure Release Ventilation or APRV or BiPAP or length of stay or LOS or absenteeism or presenteeism or work product$ or WPAI$ or productivity loss or economic burden).ti,ab.

39	exp quality of life/ or (qol or quality of life or hrql or hrqol or quality adjusted life year$ or qaly or patient reported outcome$ or satisfaction or preference$ or activities of daily living or adl or assessment of quality of life or aqol or quality of well being scale or mental health or anxiety or anxious or depress$ or psycholog$).ti,ab.
40	((utilit* and health) or (utilit* and scor*) or (utilit* and valu*) or (disutilit* and health) or (disutilit* and scor*) or (disutilit* and valu*) or daly or dalys or disability adjusted life year$ or standard gamble or time trade-off or time tradeoff or visual analog$ scale or discrete choice experiment or qwb or 15d or health utilities index or hui or hui1 or hui2 or hui3).ti,ab.
41	(sf36 or sf-36 or sf6 or sf-6 or short form 6 or sf6d or sf-6d or short form 6d or eq-5d or eq5d or euroqol or euro-qol or health status or hye or hyes or health$ year$ equivalent$ or rosser index or quality of wellbeing or qwb).ti,ab.
42	exp Health Care Costs/ or exp Drug Costs/ or exp "Cost of Illness"/ or exp Hospital Costs/ or exp Economics, Pharmaceutical/ or (treatment cost$ or direct cost$ or direct medical cost$ or nonmedical cost$ or non-medical cost$ or total cost or total costs or cost per patient treated or budget impact or cost burden or societal cost$ or administrative cost$ or travel cost$ or travel time or disease cost or cost of drugs).ti,ab.
43	exp patient isolation/ or (shield$ or isolat$).ti,ab.
44	loneliness/ or (lonely or loneliness).ti,ab.
45	physical distancing/ or ((social or physical) adj distanc$).ti,ab.
46	or/38-45
47	37 and 46
48	exp Longitudinal Studies/ or exp Retrospective Studies/ or exp Prospective Studies/ or exp Cohort Studies/ or exp Cross-Sectional Studies/ or (longitudinal study or retrospective study or prospective study or cohort$ or follow up or cross-sectional study or cross sectional study or followup study or observational study or registry or registries or real world or cross sectional or RWE).ti,ab.
49	47 and 48
50	exp book/ or exp theoretical study/ or exp case report/ or (letter or editorial or erratum or note or short survey).pt.
51	case reports.pt. or case report$.jw. or (case report or case series or woman or man or child or adolescent or female or male or boy or girl or infant).ti.
52	(Ephemera or "Introductory Journal Article" or News or "Newspaper Article" or Editorial or Comment or Overall).pt. or in vitro study/ or (commentary or editorial or comment or letter or mice or rat or mouse or animal or murine).ti.
53	exp clinical trial/ or exp randomized controlled trial/
54	or/50-53
55	49 not 54
56	limit 55 to yr="2021 -Current"

Supplemental Table 4. EconLit search strategy.

No.	Search Terms
1	(covid-19 or covid19 or corona-virus or sars-cov-2 or sars-cov2 or coronavirus disease or ncov or n-cov or delta or omicron).ti.
2	asthma$.ti.
3	(bronchiectasis or bronchiectasia or bronchoectasia).ti.
4	(cancer or neoplasm$ or leuk?emia or lymphoma or mesothelioma or myeloma or melanoma or carcinoma or adenocarcinoma or sarcoma or hemangioma or schwannoma$ or teratoma$ or tumo?r or tumo?rs).ti.
5	(brain angiopathy or brain circulation failure or brain vascular disease or brain vasculopathy or cerebral small vessel disease$ or cerebral vascular disease or cerebral vascular disorder or cerebral vascular disturbance or cerebral vascular lesion or cerebral vasculopathy or cerebrovascular damage or cerebrovascular disorder$ or cerebrovascular lesion or cerebrovascular pathology or cerebrovascular syndrome or basal ganglion h?emorrhage or bow hunter syndrome$ or brain hemangioma or brain hematoma or ((((brain or cerebral or corpus callosum or intracranial or intracerebral or periventricular or posterior fossa) adj2 (h?emorrhage or h?emorrhagic)) or ((brain or cerebral or cerebrovascular or hemisphere) adj2 infarct$) or ((brain or cerebral or cerebrovascular or cerebri or neural) adj2 (isch?emia or arterial insufficiency or circulation disorder$ or blood flow disorder or circulation disorder or circulatory disorder or encephalopathy)) or brain vasospasm or ischemic encephalopathy or transient ischemic attack or carotid artery disease$ or carotid arterial disease or carotid arteriopathy or carotid disease or (carotid artery adj2 (aneurysm or anomaly or bruit or calcification or injury or obstruction or atherosclerosis)) or moyamoya disease or cerebral artery disease$ or cerebrovascular accident or stroke or apoplexia or apoplexy or ((brain or cerebral or cerebrovascular) adj2 (accident or attack or insult or apoplexia or failure)) or cerebrovascular malformation or brain arteriovenous malformation or vein of galen malformation or hypophysis apoplexy or intracranial aneurysm or (brain or cerebral or cerebrovascular or intracranial)) adj2 (obstruction or occlusion or thrombosis or phlebothrombosis or thromboembolism or thrombosis or occlusive)) or melas syndrome or occlusive cerebrovascular disease or ocular isch?emic syndrome or posterior reversible encephalopathy syndrome or vertebrobasilar insufficiency or Wallenberg syndrome).ti.
6	(((chronic kidney or renal) adj2 (disease$ or disorder$ or insufficien$ or fail$)) or ((kidney or renal) adj2 (transplant$ or graft$ or allograft$))).ti. or (CKF or CKD or CRF or CRD or ESRF or ESKF or ESKD or ESRD or CAPD or CCPD or APD).ti,ab. or (renal replacement$ or hemodialysis or haemodialysis or hemofiltration or haemofiltration or hemodiafiltration or haemodiafiltration or predialysis or pre-dialysis or dialysis).ti.
7	((solid organ or kidney or heart or liver or intestin$ or lung or pancreas or hematopoietic stem cell$ or blood stem cell$ or bone marrow) adj5 transplant$).ti.
8	(primary immunodeficienc$ or primary immune deficienc$ or diGeorge or wiskott-aldrich or PID or PIDDs).ti.
9	chronic liver disease.ti.
10	cirrhosis.ti.
11	(NASH or ((nonalcoholic or non-alcoholic) adj3 (steatohepatitis or fatty liver))).ti.
12	(alcoholic liver disease$ or alcoholic liver disorder$).ti.
13	(autoimmune hepatitis or lupoid hepatitis).ti.
14	(chronic airflow obstruction$ or chronic airway obstruction$ or chronic obstructive bronchopulmonary disease$ or chronic obstructive lung disorder$ or chronic obstructive pulmonary disease$ or chronic obstructive pulmonary disorder$ or chronic obstructive respiratory disease$ or chronic pulmonary obstructive disease$ or chronic pulmonary obstructive disorder$ or copd$ or lung chronic obstructive disease$ or obstructive chronic lung disease$ or obstructive chronic pulmonary disease$).ti.
15	(cystic fibrosis or cystic pancreas fibrosis or cystic pancreatic fibrosis or fibrocystic disease or mckusick 21970 or mucoviscidos?s or pancreas cystic disease or pancreas cystic fibrosis or pancreas fibrocystic disease or pancreas fibrosis or pancreatic cystic disease or pancreatic cystic fibrosis or pancreatic fibrosis).ti.
16	(type 1 diabet$ or type I diabet$ or t1dm or insulin-dependent diabet$ or IDDM).ti.
17	(type 2 diabet$ or type II diabet$ or t2dm or non-insulin-dependent diabet$ or noninsulin-dependent diabet$ or NIDDM).ti.
18	(down syndrome$ or trisomy 13$).ti.
19	(Disabilit$ or disabled or (impair* adj2 (physical* or visual$ or vision$ or hear$ or sensory$)) or blind or deaf or handicap$ or cerebral palsy or autism or autistic or asperger$ or ADHD or

	Trisomy or Fragile X or Muscular Dystroph$ or Tourette$ or Epilep$ or seizure$ or Neuropath$ or neuromuscular or myasthenia$).ti.
20	(cardiac backward failure or cardiac decompensation or cardiac failure or cardiac incompetence or cardiac insufficienc$ or cardiac stand still or cardial decompensation or cardial insufficienc$ or chronic heart insufficienc$ or decompensatio cordis or heart backward failure or heart decompensation or heart failure or heart incompetence or heart insufficienc$ or insufficientia cardis or myocardial failure or myocardial insufficienc$ or CHF or HFREF or HFPEF).ti.
21	(acute coronary syndrome$ or acs or Myocardial Infarction$ or AMI or STEMI or NSTEMI or non-stemi or Unstable Angina or Myocardial preinfarct$ or Myocardial pre-infarct$ or Preinfarct angina or pre-infarct angina or preinfarction angina or pre-infarction angina or Angina at rest or Variant angina or Prinzmetals angina or cardiac allograft vasculopathy or (coronary artery adj2 (aneurysm or anomaly or atherosclerosis or calcification or constriction or dissection or obstruction or occlusion or perforation or thrombosis)) or coronary bifurcation lesion or coronary subclavian steal syndrome or kounis syndrome or no reflow phenomenon).ti.
22	(cardiomyopathy or barth syndrome or chagas or heart amyloidosis or heart right venticle dysplasia or kearns sayre syndrome or ventricular noncompaction).ti.
23	(HIV or human immunodeficiency virus).ti.
24	(diffuse interstitial pneumopath$ or diffuse parenchyma lung disease$ or diffuse parenchymal lung disease or diffuse parenchymal pulmonary disease$ or diffuse parenchymal pulmonary disorder or interstitial lung disease$ or interstitial lung disorder$ or interstitial pneumopathy or interstitial pulmonary disease or interstitial pulmonary disorder or antisynthetase syndrome or berylliosis or fibrosing alveolitis or interstitial pneumonia or loeffler pneumonia or pneumoconiosis or wegener granulomatosis).ti.
25	(((mood or affective) adj2 (disorder$ or disturbance or illness)) or affective neurosis or affective psychosis or blunted affect or depression$ or depressive or bipolar or dysphoria or dysthymia or melancholia or perry syndrome or premenstrual dysphoric disorder or pseudodementia or seasonal affective disorder or major affective disorder or mania or hypomania or manic$ or minor affective disorder or schizoaffective psychosis).ti.
26	(schizophrenia or schizophrenic or dementia praecox).ti.
27	(neurocognitive disorder$ or cognitive impairment$ or dementia$ or amentia$).ti.
28	(obesity or adipose tissue hyperplasia or adipositas or adiposity or excess body weight or corpulency or fat overload syndrome or obesitas or overweight or lipedema or metabolic syndrome).ti.
29	physical inactivit$.ti.
30	(pregnan$ or gestation or gravidity or child-bearing or childbearing or post-partum or postpartum or puerperium or puerperal or weaning).ti.
31	(pulmonary arterial hypertension or pulmonary artery hypertension or lung artery hypertension or lung arterial hypertension or lung hypertension or pulmonary hypertensive disease or pulmonary hypertensive disorder or pulmonary hypertension or pulmonary embol$ or lung embol$ or lung microembol$ or pulmonary thromboembol$).ti.
32	(smoking or smoker$ or "tobacco use" or non-smoker$ or ex-smoker$ or never-smoker$ or nonsmoker$).ti.
33	(tuberculosis or tuberculous).ti.
34	(steroid$ or corticosteroid$ or abiraterone acetate or androstane derivative or azasteroid or catatoxic steroid or chandonium iodide or clascoterone or diosgenin or estrane derivative or etiocholanolone or fluasterone or ganaxolone or gonane derivative or hecogenin or homosteroid or hydroxysteroid or mipicoledine or neurosteroid or norsteroid or oxosteroid or pamaqueside or pregnane derivative or rocuronium or rostafuroxin or sarsasapogenin or secosteroid or smilagenin or sonolisib or spirostan derivative or brassinolide or nassinosteroid or casasterone or ecdysone or ecdysteroid or ecdysterone or ponasterone A or hydroxycorticosteroid or henzodrocortisone or glucocorticoid or alclometasone or algestone or amcinonide or amelometasone or beclometasone or betamethasone or celestamine or budesonide or butixocort or (chlorhexidine acetate and tixocortol pivalate) or chloroprednisone or (chlorpheniramine maleate and cortisone acetate) or (chlorquinaldol and promestriene) or ciclesonide or ciprocinonide or (ciprofloxacin and fluocinolone acetonide) or (clioquinol and flumetasone pivalate) or clobetasol or clobetasone or clocortolone or cloprednol or cortisone or tetrahydrocortisone or cortivazol or deflazacort or dexamethasone or baycuter or desoximetasone or dexatopic or sofradex or diflorasone or diflucortolone or difluprednate or domoprednate or drocinonide or dutimelan or etiprednol dicloacetate or fluclorolone or fludrocortisone or fludroxycortide or flumetasone or flumoxonide or flunisolide or fluocinolone or fluocinonide or fluocortin or fluocortolone or fluorometholone or fluprednidene or fluprednisolone or fluticasone or

	formocortal or halcinonide or halometasone or halopredone or hydrocortisone or epihydrocortisone or hydrasllostane or hydrocortamate or tetrahydrocortisol or icometasone enbutate or isoflupredone or itrocinonide or locicortolone dicibate or lorinden-a or lorinden-t or loteprednol or mazipredone or medrysone or meprednisone or mometasone furoate or nicocortonide or nivacortol or oropivalone or paramethasone or prednisolone or prednisone or pregnenolone or procinonide or promestriene or resocortol or rimexolone or rofleponide or ticabesone or timobesone or tipredane or tixocortol or triamcinolone or mycolog or ulobetasol propionate or uniderm or vamorolone or zoticasone or mineralocorticoid or aldosterone or corticosterone or dehydrocorticosterone or deoxycorticosterone or tetrahydroeoxycorticosterone or fludrocortisone or vecuronium or zuranolone).ti.
35	(immunosuppress$ or abatacept or abetimus or aldophosphamide or alemtuzumab or amsacrine or anifrolumab or anisperimus or apilimod or ascomycin or ascrolimus or aselizumab or atacicept or atorolimumab or avacopan or avizakimab or azathioprine or basiliximab or batoclimab or batriden or beclomethasone dipropionate or begelomab or belatacept or belimumab or betamethasone dipropionate or blisibimod or brepocitinib or briobacept or carisoprodol plus prednisolone or cedelizumab or cendakimab or cenerimod or cenplacel-L or certolizumab pegol or cm-4620 or CM4620 or colchicine or cusatuzumab or cyclophosphamide or cyclosporin$ or daclizumab or dafsolimab setaritox or darvadstrocel or daxdilimab or dazodalibep or deflazacort or defoslimod or dehydrodidemnin B or dexamethasone or didemnin-A or didemnin-B or dihydrocyclosporin A or dimethyl fumarate or dorlimomab aritox or ebdarokimab or ect-001 or edratide or efalizumab or eldelumab or elsilimomab or elsubrutinib or emapalumab or enlimomab or enlimomab pegol or erlizumab or etrasimod or faralimomab or fezakinumab or filgotinib or fingolimod or fontolizumab or forigerimod or forodesine or fosifidancitinib or fr-900523 or fr900523 or fr-900525 or fr900525 or glatiramer or govitecan or grisnilimab setaritox or guselkumab or ifidancitinib or imilecleucel-t or imlifidase or inebilizumab or inolimomab or interleukin-2 receptor antibody or irinotecan or izencitinib or laflunimus or lazucirnon or lebrikizumab or leflunomide or lerociclib or letolizumab or levilimab or lirentelimab or ljp-1082 or lusvertikimab or malononitrilamide or manitimus or maslimomab or merimepodib or methotrexate or mizoribine or mocravimod or morolimumab or mycophenolate mofetil or mycophenolic acid or napirimus or narsoplimab or natalizumab or nimacimab or nipocalimab or obexelimab or ocrelizumab or ofatumumab or OKT-3 or olamkicept or olcorolimus or olendalizumab or omalizumab or orazipone or oxeclosporin or ozanimod or paquinimod or pascolizumab or pateclizumab or peldesine or perfosfamide or pimecrolimus or plovamer or pn-1007 or Polypodium leucotomos extract or ponesimod or pritoxaximab or prodigiosin or ramatercept or rapamycin or rb-212 or rb212 or relfovetmab or remestemcel-L or reprimun or reproxalap or rilonacept or rilzabrutinib or rimiducid or risankizumab or rlyb211 or rozanolixizumab or ruclosporin or sanglifehrin-A or sonelokimab or sotrastaurin or sufosfamide or suppressor factor or tacrolimus or telimomab aritox or temsirolimus or teneliximab or teplizumab or thermozymocidin or tiplimotide or tocilizumab or tofacitinib or tol2 or traxanox or tresperimus or trilaciclib or tumor necrosis factor inhibitor$ or TNF-inhibitor$ or adalimumab or amlitelimab or belantamab or bleselumab or CD24Fc or cudarolimab or denosumab or efizonerimod alfa or etanercept or giloralimab or golimumab or infliximab or mitazalimab or pavurutamab or pegilodecakin or peimine or quellor or ravagalimab or remtolumab or selicrelumab or sibeprenlimab or sotigalimab or tanfanercept or tavolimab or telazorlimab or telitacicept or tibulizumab or umirolimus or valziflocept or vapaliximab or vepalimomab or vgx-1027 or vgx1027 or vidofludimus or voclosporin or zolimomab aritox or zotarolimus).ti.
36	or/2-35
37	1 and 36
38	limit 37 to yr="2021 -Current"

Supplemental Table 5. Base immunocompromised population considered in this review article.

- Individuals with solid tumors or hematologic malignancies, including chronic lymphocytic leukemia
- Individuals undergoing CAR-T therapy or recent hematopoietic stem cell transplant (ie, within 2 years of transplantation or undergoing immunosuppression therapy)
- Solid organ transplant recipient and undergoing immunosuppressive therapy
- Active treatment with:
    - High-dose corticosteroids (ie, ≥20 mg prednisone or equivalent per day when administered for ≥2 weeks)
    - Alkylating agents
    - Antimetabolites
    - Transplant-related immunosuppressive drugs
    - Cancer chemotherapeutic agents classified as severely immunosuppressive
    - TNF inhibitors
- Advanced or untreated HIV (ie, not suppressed)
- Moderate or severe primary immunodeficiency disorder (such as DiGeorge syndrome or Wiskott-Aldrich syndrome)
- CKD or ESRD who are undergoing dialysis

CAR chimeric antigen receptor, *CKD* chronic kidney disease, *ESRD* end-stage renal disease, *HIV* human immunodeficiency virus, *TNF* tumor necrosis factor

Supplemental Table 6. Healthcare resource utilization and mental health results during early pandemic.

Healthcare resource utilization: Early pandemic (early to mid-2020): pre-vaccination						
**Publication/study date/country**	**Population**	**Vaccination status**	**Hospitalizations, *n* (%)**	**ICU admissions, *n* (%)**	**Mechanical ventilation, *n* (%)**	**Risk factor group comparisons**
Lievre et al., 2020<br>Apr–Jun 2020<br>France[1]	People with solid tumors and COVID-19<br>(*n* = 1289)	Pre-vaccination	734 (65)	110 (10)	49 (5)	COVID-19 severity<br>• Corticosteroids before COVID-19 diagnosis (ref: no) (OR, 1.60 [95% CI, 1.03–2.48]; *P* = .04)<br>• Thoracic primary tumor location (ref: others) (OR, 1.50 [95% CI, 1.02–2.21]; *P* = .04)<br>• ECOG PS ≥2 (ref: 0–1) (OR, 2.61 [95% CI, 1.88–3.62]; *P* < 0.0001)<br>• Updated Charlson Comorbidity Index (OR, 1.08 [95% CI, 1.02–1.15]; *P* = .0147)
Crolley et al., 2020<br>Mar–Jul 2020<br>UK[2]	Adults with cancer receiving systemic anti-cancer therapy and COVID-19<br>(*n* = 68)	Pre-vaccination	60 (88)	–	–	–
Ruiz-Garcia et al., 2021<br>May–Dec 2020<br>Mexico[3]	Adults with cancer and COVID-19<br>(*n* = 599)	Pre-vaccination	248 (73)	–	57 (23)	-
Serraino et al., 2021<br>Feb–Dec 2020<br>Italy[4]	Adults with cancer and positive for COVID-19<br>(*n* = 3098)	Pre-vaccination	–	93 (3)[a]	–	ICU admission (COVID-19 positive vs COVID-19 negative)<br>• Aged 70–79 years (HR, 1.44 [95% CI, 1.06–1.97])<br>• Respiratory disease (HR, 1.80 [95% CI, 1.18–2.73])<br>• Diagnosed with cancer >60 months prior to COVID-19 (HR, 1.38 [95% CI, 1.02–1.86])<br>• Hematological malignancy and COVID-19 (HR, 1.95 [95% CI, 1.02–3.75])
	Adults with cancer and negative for COVID-19<br>(*n* = 23,296)		–	960 (4)[a]	–	
	Adults without cancer and positive for COVID-19<br>(*n* = 38,268)		–	502 (1)[a]	–	
Wang et al., 2020<br>Start of pandemic–Aug 2020<br>USA[5]	Adults with recent cancer and COVID-19<br>(*n* = 670)	Pre-vaccination	320 (48)[a]	–	–	Hospitalization rate<br>• Recent cancer diagnosis and COVID-19 vs COVID-19 but no recent cancer diagnosis (*P* < .001)<br>• Recent cancer diagnosis and COVID-19 vs recent cancer but no COVID-19 (*P* < .001)
	Adults with recent cancer<br>(*n* = 270,380)		33,490 (12)[a]	–	–	
	Adults with COVID-19 (no recent cancer)<br>(*n* = 14,840)		3600 (24)[a]	–	–	
Costa et al., 2021<br>Mar–Dec 2020<br>Brazil[6]	Adults with cancer hospitalized with COVID-19<br>(*n* = 7406)	Pre-vaccination	All patients in study	3159 (45)	1802 (27)	ICU admission rate<br>• Cancer vs no cancer (*P* < .001)

	Adults without cancer hospitalized with COVID-19<br>(*n* = 315,410)		All patients in study	113,481 (40)	60,197 (22)	
Fillmore et al., 2021<br>Jan–May 2020<br>USA[7]	Veterans with cancer and COVID-19<br>(*n* = 1794)	Pre-vaccination	N/A (43.8; 12.3 due to COVID-19)	N/A (19.7; 11.8 due to COVID-19)	N/A (7.9; 6.6 due to COVID-19)	-
	Veterans with cancer (no COVID-19)<br>(*n* = 21,120)		N/A (32)	N/A (8)	N/A (1)	
Roel et al., 2022<br>Mar–May 2020<br>Spain[8]	Adults with cancer and COVID-19<br>(*n* = 5393)	Pre-vaccination	735 (14)	–	–	Hospitalization<br>• Cancer (vs no cancer) (aHR, 1.33 [95% CI, 1.24–1.43])<br>• Recent cancer (<1 year since diagnosis) (ref: no cancer) (aHR, 1.84 [95% CI, 1.52–2.23])<br>• Hematological cancer (<1 year since diagnosis) (ref: no cancer) (aHR, 6.18 [95% CI, 4.31–8.86])<br>• Cancer (<5 years since diagnosis) (ref: no cancer) breast (aHR, 1.12 [95% CI, 0.79–1.56]), colorectal (aHR, 1.09 [0.81–1.47]), and bladder (aHR, 0.78 [0.51–1.20])
	Adults with COVID-19 (no cancer)<br>(*n* = 93,558)		6116 (7)	–	–	
Rugge et al., 2023<br>Feb–Sep 2020<br>Italy[9]	Adults with cancer and COVID-19<br>(*n* = 1787)	Pre-vaccination	713 (40)	89 (5)	–	Hospitalization<br>• Cancer diagnosis ≤12 months before COVID-19 (ref: no history of cancer) (OR, 2.88 [95% CI, 2.22–3.76])<br>• Lung cancer (ref: no cancer) (OR, 4.52 [95% CI, 2.44–8.72])<br>• Hematological cancer (ref: no cancer) (OR, 3.01 [95% CI, 2.16–4.21])
	Adults with COVID-19 (no cancer)<br>(*n* = 20,777)		4685 (23)	734 (4)	–	
Kwon et al., 2022<br>Feb–Dec 2020<br>USA[10]	Adults with cancer and COVID-19<br>(*n* = 1781)	Pre-vaccination	388 (22)*	–	–	Hospitalization<br>• Myeloproliferative neoplasm (vs unspecified cancer type) (RR, 2.15 [95% CI, 1.25–3.41])<br>• Non-chemotherapy targeted cancer therapy (vs not) (RR, 2.82 [95% CI, 1.19–5.19]; *P* = .021)<br>• Methotrexate (RR, 2.72 [95% CI, 1.10–5.19]; *P* = .032)<br>• Venetoclax (RR, 2.96 [95% CI, 1.14–5.66]; *P* = .028)
Pinato et al., 2022<br>Feb–Nov 2020<br>Europe[11]	Adults with cancer and COVID-19<br>(*n* = 2033)	Pre-vaccination	1142 (57)	–	–	

**Mental health: Early pandemic (early to mid-2020): pre-vaccination**						
**Publication/study date/country**	**Population**	**Vaccination/ infection status**	**Domain**	**Instrument**	**Comparison**	**Mental health results**
Lamblin et al., 2022<br>Mar 2020–Sep 2020<br>France[12]	Adults with non-metastatic breast or gynecological cancer<br>(*n* = 125)	• Unvaccinated<br>• COVID-19: 3.4%	Depression	HADS	Lockdown vs post-lockdown	• Mean depression score was significantly lower post-lockdown vs during lockdown
			Anxiety			• Mean anxiety score was lower post-lockdown vs during lockdown, but not significantly so

**Mental health: Early pandemic (early to mid-2020): pre-vaccination**						
Ellehuus et al., 2021<br>May 2020–Jun 2020<br>Denmark[13]	Adults with hematological cancer (*n* = 2239)	• Unvaccinated<br>• Infection status unknown	Anxiety	GAD-7	None	• Symptoms of generalized anxiety associated with COVID-19: 20%<br>○ Low symptoms of anxiety: 79%<br>○ Mild symptoms of anxiety: 16%<br>○ Moderate to severe symptoms of anxiety: 5%<br>• Significantly associated with mild-to-moderate anxiety: being female and having received medical treatment or blood transfusion <2 months prior
Marino et al., 2022<br>Jun 2020<br>France[14]	Adults with any cancer (*n* = 1097)	• Unvaccinated<br>• COVID-19: 7.7%	Anxiety	STAI<br>(*n* = 962 respondents)	None	• Anxiety: 31%<br>• Fear of cancer recurrence main predictor of anxiety
			Distress	IES-R<br>(*n* = 810 respondents)		• Low level PTSD: 85%<br>• Moderate-severe PTSD: 15%<br>• Fear of going to hospital due to risk of contracting COVID-19 strongest predictor of PTSD<br>• Better lockdown experience was protective against PTSD
Graves et al., 2022<br>May 2020–Oct 2020<br>USA[15]	Adults with thyroid cancer (*n* = 378)	• Unvaccinated<br>• COVID-19: 18.8%	Anxiety<br>Depression	PROMIS-29	Pre-COVID vs COVID era	• Levels of anxiety and depression significantly higher during vs pre-pandemic (*P* < .05)<br>• Younger age and change in treatment plan significantly associated with increased anxiety
Ye et al., 2022<br>Feb 2020–Mar 2020<br>China[16]	People with cancer (*n* = 1003) and people without cancer (*n* = 185)	• Unvaccinated<br>• Infection status unknown	Distress	IES-R	Patients with cancer vs without cancer	• Treatment delays associated with increased PTSD<br>• Good communication with doctor associated with decreased PTSD (vs unsatisfactory or no communication)
Borsari et al., 2022<br>Mar 2020–May 2020<br>Italy[17]	Adults referred for skin cancer (*n* = 355)	• Unvaccinated<br>• Infection status unknown	Distress	IES-R	Pre- vs during lockdown	• Distress levels significantly increased from pre-lockdown to lockdown

[a]Outcomes not explicitly specified as COVID-19 related.
Shading indicates poorer mental health during pandemic/in people with cancer versus relevant comparator.
aHR, adjusted hazard ratio; aIRR, adjusted incremental rate ratio; aOR, adjusted odds ratio; CI, confidence interval; CLL, chronic lymphocytic leukemia; ECOG PS, Eastern Cooperative Oncology Group performance status; GAD-7, General Anxiety Disorder-7; HADS, Hospital Anxiety and Depression Scale; HR, hazard ratio; ICU, intensive care unit; IES-R, Impact of Event Scale - Revised; MV, mechanical ventilation; N/A, not available; OR, odds ratio; PROMIS-29, Patient-Reported Outcomes Measurement Information System-29; PTSD, post-traumatic stress disorder; RR, rate ratio.

References

1. Lièvre A, Turpin A, Ray-Coquard I, et al. Risk factors for coronavirus disease 2019 (COVID-19) severity and mortality among solid cancer patients and impact of the disease on anticancer treatment: a French nationwide cohort study (GCO-002 CACOVID-19). *Eur J Cancer.* 2020;141:62-81. https://doi.org/10.1016/j.ejca.2020.09.035.
2. Crolley VE, Hanna D, Joharatnam-Hogan N, et al. COVID-19 in cancer patients on systemic anti-cancer therapies: outcomes from the CAPITOL (COVID-19 Cancer PatIenT Outcomes in North London) cohort study. *Ther Adv Med Oncol.* 2020;12:1758835920971147. https://doi.org/10.1177/1758835920971147.
3. Ruiz-Garcia E, Pena-Nieves A, Alegria-Banos J, et al. Prognostic factors in cancer patients infected with SARS-CoV-2: a Latin American country results. *Ther Adv Chronic Dis.* 2021;12:20406223211047755. https://doi.org/10.1177/20406223211047755.
4. Serraino D, Zucchetto A, Dal Maso L, et al. Prevalence, determinants, and outcomes of SARS-COV-2 infection among cancer patients. A population-based study in northern Italy. *Cancer Med.* 2021;10(21):7781-7792. https://doi.org/10.1002/cam4.4271.
5. Wang QQ, Berger NA, Xu R. Analyses of risk, racial disparity, and outcomes among US patients with cancer and COVID-19 infection. *JAMA Oncol.* 2021;7(2):220-227. https://doi.org/10.1001/jamaoncol.2020.6178.
6. Costa GJ, de Azevedo C, Junior JIC, et al. Higher severity and risk of in-hospital mortality for COVID-19 patients with cancer during the year 2020 in Brazil: a countrywide analysis of secondary data. *Cancer.* 2021;127(22):4240-4248. https://doi.org/10.1002/cncr.33832.
7. Fillmore NR, La J, Szalat RE, et al. Prevalence and outcome of COVID-19 infection in cancer patients: a National Veterans Affairs study. *J Natl Cancer Inst.* 2021;113(6):691-698. https://doi.org/10.1093/jnci/djaa159.
8. Roel E, Pistillo A, Recalde M, et al. Cancer and the risk of coronavirus disease 2019 diagnosis, hospitalisation and death: a population-based multistate cohort study including 4 618 377 adults in Catalonia, Spain. *Int J Cancer.* 2022;150(5):782-794. https://doi.org/10.1002/ijc.33846.
9. Rugge M, Zorzi M, Guzzinati S, et al. Outcomes of SARS-CoV-2 infection in cancer versus non-cancer-patients: a population-based study in northeastern Italy. *Tumori.* 2023;109(1):38-46. https://doi.org/10.1177/03008916211073771.
10. Kwon DH, Cadena J, Nguyen S, et al. COVID-19 outcomes in patients with cancer: findings from the University of California health system database. *Cancer Med.* 2022;11(11):2204-2215. https://doi.org/10.1002/cam4.4604.
11. Pinato DJ, Aguilar-Company J, Ferrante D, et al. Outcomes of the SARS-CoV-2 Omicron (B.1.1.529) variant outbreak among vaccinated and unvaccinated patients with cancer in Europe: results from the retrospective, multicentre, OnCovid registry study. *Lancet Oncol.* 2022;23(7):865-875. https://doi.org/10.1016/S1470-2045(22)00273-X.
12. Lamblin G, Chene G, Leaune E, et al. The psychological impact of therapeutic changes during the COVID-19-lockdown for gynaecological and breast cancer patients. *J Gynecol Obstet Hum Reprod.* 2022;51(3):102311. https://doi.org/10.1016/j.jogoh.2022.102311.
13. Ellehuus C, Jeppesen SS, Eckhoff L, et al. Health-related quality of life of patients with haematologic cancer during COVID-19 and their opinions on telehealth consultations–a Danish single site cross-sectional survey. *Acta Oncologica.* 2021;60(7):872-880. https://doi.org/10.1080/0284186X.2021.1902565.
14. Marino P, Touzani R, Pakradouni J, et al. The psychological distress of cancer patients following the COVID-19 pandemic first lockdown: results from a large French survey. *Cancers (Basel).* 2022;14(7). https://doi.org/10.3390/cancers14071794.

15. Graves CE, Goyal N, Levin A, et al. Anxiety during the COVID-19 pandemic: a web-based survey of thyroid cancer survivors. *Endocr Pract.* 2022;28(4):405-413. https://doi.org/10.1016/j.eprac.2022.01.002.
16. Ye Y, Wang J, Cai S, et al. Psychological distress of cancer patients caused by treatment delay during the COVID-19 pandemic in China: a cross-sectional study. *Psychooncology.* 2022;31(9):1607-1615. https://dx.doi.org/10.1002/pon.5946.
17. Borsari S, Pampena R, Benati M, et al. Self-reported measure of subjective distress in response to COVID-19 pandemic in patients referred to our skin cancer unit during the first wave. *Clin Dermatol.* 2022;40(1):93-99. https://doi.org/10.1016/j.clindermatol.2021.11.014.
